# Outer membrane vesicles as a versatile nanoplatform for advanced vaccine development and immunotherapy

**DOI:** 10.3389/fimmu.2026.1707391

**Published:** 2026-01-27

**Authors:** Jiawen Zhang, Yuhang Zhi, Yu Lu, Fang Ma

**Affiliations:** 1 Institute of Veterinary Immunology & Engineering, National Research Center of Engineering and Technology for Veterinary Biologicals, Jiangsu Academy of Agricultural Sciences, Nanjing, China; 2 College of Veterinary Medicine, Nanjing Agricultural University, Nanjing, China; 3 China-South Africa Joint Laboratory For Prevention and Control of Major Animal Diseases, Nanjing, China

**Keywords:** bacterial outer membrane vesicle, bioengineering applications, immune modulation, nanoscale vesicles, target delivery

## Abstract

Bacterial outer membrane vesicles (OMVs) are nano-sized, lipid-bilayer vesicles naturally released by Gram-negative bacteria. These vesicles are enriched with diverse biomolecules, including lipids, proteins, and nucleic acids, enabling them to mediate critical biological processes. Emerging evidence highlights their pivotal roles in bacterial physiology, intercellular communication, and environmental adaptation, alongside their promising therapeutic potential. This review focuses on recent advances in OMV biogenesis, composition, function and applications. By integrating current knowledge, we aim to inspire novel insights into the molecular mechanisms underlying OMV functions and facilitate their translational development in medicine. Ultimately, this work serves as a comprehensive reference to accelerate future research and clinical utilization of this versatile platform.

## 1 Introduction

The initial discovery of bacterial outer membrane vesicle (OMVs) dates to the 1960s, when electron microscopy first revealed their presence in Gram-negative bacteria (1). Initially dismissed as cellular debris due to limited understanding of their biological roles, OMVs gained scientific attention following their detection in the plasma of a patient with fatal meningococcal septicemia (2). As research advanced, these vesicular structures were identified in an expanding range of bacterial species, including Gram-positive organisms (3).

OMVs are nano-sized with diameters ranging from 20-300 nm, spherical structures naturally released by Gram-negative bacteria through outer membrane (OM) budding (4). Their characteristic components, including lipopolysaccharide (LPS), outer membrane proteins (OMPs), and associated peptidoglycan (PG), vary significantly across bacterial species and growth conditions, directly correlating with functional diversity (5, 6). For bacteria, OMVs multifunctional tools central to survival and adaptation. They serve as communication vehicles, facilitating interbacterial signaling, horizontal gene transfer (HGT), and virulence factor dissemination (7–9). Simultaneously, they act as a defense system by neutralizing antimicrobial compounds and as an offensive weapon by delivering cytotoxic effectors to host cells (10, 11). Furthermore, OMVs are integral to biofilm formation, providing structural support and enhancing community resilience (12). In host interactions, OMVs directly modulate immune responses, capable of both activating and suppressing specific pathways (13). This complex interplay between microbial activity and host immunomodulation underscores their significance in infection. The convergence of these biological properties has propelled OMVs into biomedical applications. Their natural nanostructure and biocompatibility make them excellent drug delivery platforms, while their immunogenic and modifiable surfaces position them as novel vaccine candidates and sensitive diagnostic tools (14, 15).

Compared to other nanoparticle and extracellular vesicle platforms, OMVs possess distinct advantages for biomedical applications. Unlike synthetic liposomes, which require complex chemical synthesis and lack inherent immunostimulatory properties, OMVs are naturally produced with intrinsic immunogenic components, like LPS and OMPs, that can be harnessed for vaccine development (16). In contrast to mammalian-derived exosomes, which face challenges in large-scale production, batch-to-batch variability, and complex isolation procedures, OMVs can be produced in substantial quantities through bacterial fermentation with greater reproducibility and cost-effectiveness (17). Furthermore, OMVs offer superior genetic and surface engineering capabilities compared to exosomes, allowing precise modification of cargo and surface antigens through established bacterial genetic manipulation techniques (18). While synthetic nanoparticles provide controlled physicochemical properties, OMVs combine the benefits of biological origin, including natural biocompatibility, membrane fusion capacity, and pathogen-associated molecular patterns, with the scalability and modifiability typically associated with engineered systems (19). These unique characteristics position OMVs as a promising hybrid platform that bridges the gap between synthetic and naturally-derived delivery systems.

This review provides a systematic examination of the contemporary understanding of OMV biogenesis, molecular composition, and functional diversity, while offering a critical evaluation of their emerging applications in therapeutics and diagnostics. We identify and discuss pivotal challenges in OMV production standardization, safety optimization, and clinical translation. Importantly, we underscore persistent and significant knowledge gaps concerning the fundamental mechanisms of OMV biogenesis and their multifaceted roles in microbial ecology and infection biology. Addressing these gaps through focused investigation is crucial to fully unlocking the transformative biomedical potential of OMVs.

## 2 Mechanisms of OMV production and secretion by bacteria

**Figure 1:**
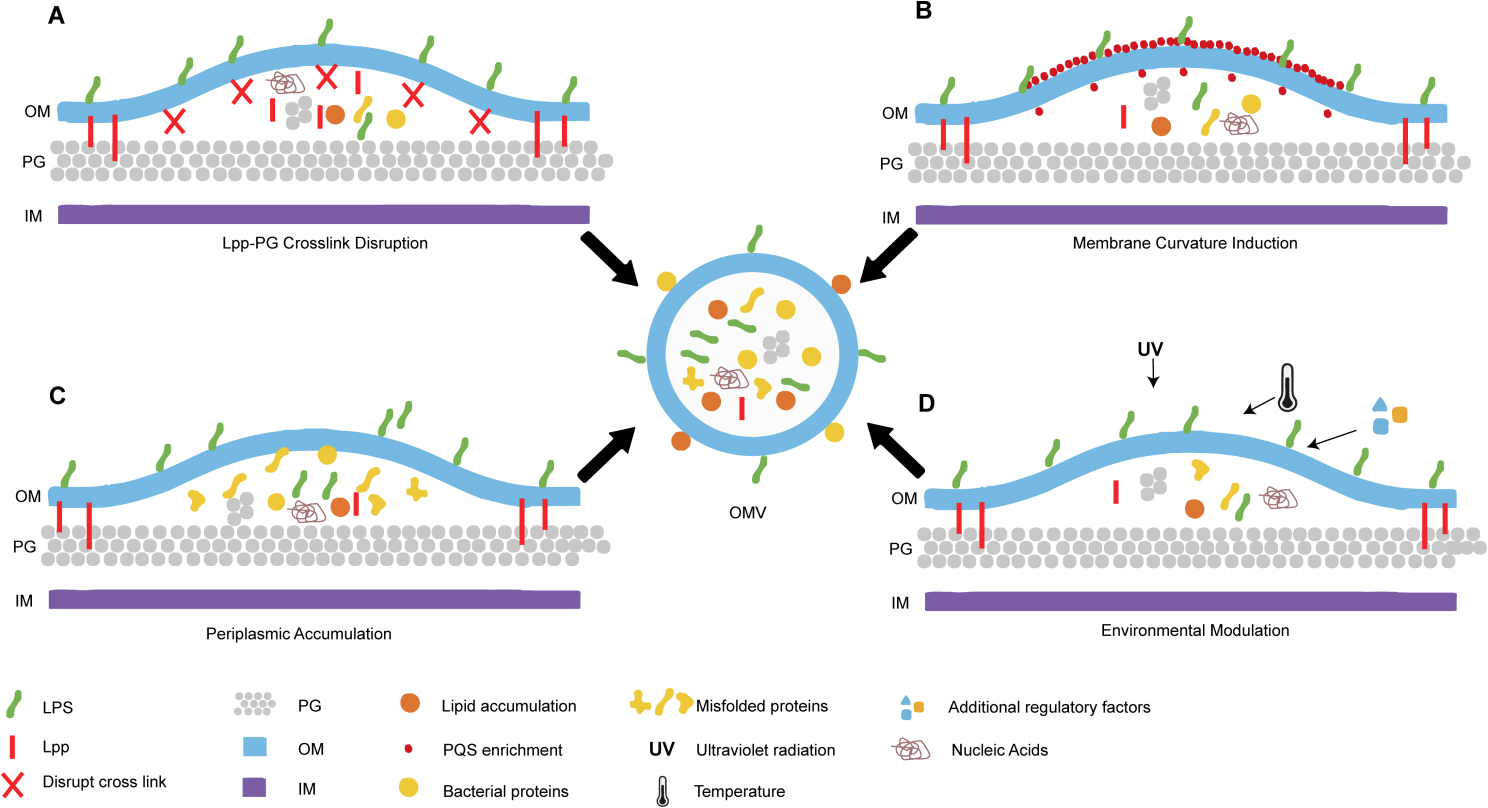
Schematic summary of bacterial outer membrane vesicles (OMVs) biogenesis. **(A)** Disruption of lipoprotein-peptidoglycan (Lpp-PG) crosslinks promotes outer membrane (OM) bulging and vesiculation. **(B)** Asymmetric Pseudomonas quinolone signal (PQS) in the OM leaflet induces curvature. **(C)** Periplasmic accumulation of misfolded proteins or lipids generates mechanical pressure. **(D)** Environmental factors modulate OMV biogenesis. Center: OMV budding from OM with cargo. Key components are labeled: Lpp, Lipoproteins; Lpp-PG, lipoprotein-peptidoglycan; PG, peptidoglycan; OM, outer membrane; IM, inner membrane; PQS, Pseudomonas quinolone signal.

OMV biogenesis occurs under diverse growth conditions and has been observed both *in vivo* and in various *in vitro* environments, including liquid cultures, solid media, biofilms, and host cell coculture systems. The fundamental process involves localized membrane blebbing, where outward curvature of the OM leads to constriction and eventual vesicle release. Although the precise regulatory mechanisms remain incompletely characterized, current evidence indicates that OMV production is a highly regulated biological process, not a stochastic event. Recent biochemical and genetic approaches have elucidated three predominant models currently debated in the literature: the lipoprotein depletion model, the envelope stress response model, and the periplasmic accumulation model. Each proposing distinct molecular triggers for initiating membrane curvature and vesicle pinching-off. A schematic summary of OMV biogenesis is presented in **Figure 1**.

### 2.1 Disruption of OM peptidoglycan crosslinking

The Gram-negative cell envelope consists of an asymmetric OM, a cytoplasmic inner membrane (IM), and a periplasmic space containing a thin PG layer (20). Structural integrity of this envelope is maintained through covalent linkages between abundant outer membrane lipoproteins (Lpp) and the PG layer. Environmental stressors can induce proteolytic cleavage of Lpp, disrupting these critical crosslinks. This destabilization promotes localized OM bulging, leading to vesicle formation via membrane curvature. Schwechheimer and colleagues provided key evidence for this mechanism by demonstrating an inverse correlation between Lpp-PG crosslinking density and OMV production, establishing that reduced tethering forces directly facilitate OMV biogenesis (6).

### 2.2 Membrane curvature induction via molecular enrichment

A biophysical model of OMV biogenesis, established by the Florez group, centers on the membrane-remodeling properties of Pseudomonas quinolone signal (PQS), 2-heptyl-3-hydroxy-4-quinolone, in *Pseudomonas aeruginosa* (*P. aeruginosa*) (21). As a quorum-sensing molecule, PQS preferentially accumulates in the outer leaflet of the OM, creating asymmetric lateral expansion relative to the inner leaflet. This molecular crowding generates an interleaflet tension differential that drives membrane curvature, the critical initial step in OMV budding (22). Current evidence indicates PQS promotes vesiculation through two synergistic mechanisms: chelating cations to disrupt stabilizing bridges between B-band LPS molecules, and enhancing electrostatic repulsion among the negatively charged LPS moieties (23). Although the PQS model represents one of the most mechanistically characterized pathways, its applicability remains phylogenetically constrained to *P. aeruginosa*. This limitation underscores the need to identify analogous curvature-inducing molecules in other Gram-negative species that may employ distinct molecular strategies for membrane remodeling and OMV production.

### 2.3 Periplasmic accumulation-induced vesiculation

The periplasmic compartment serves as another critical site for OMV biogenesis. Accumulation of misfolded proteins or peptidoglycan fragments in this confined space, due to stressors like temperature shifts or oxidative stress, generates substantial turgor pressure against the outer membrane. This mechanical stress initiates a cascade: compromising membrane integrity by disrupting OM-PG tethering, creating areas of local detachment, and ultimately driving vesicle formation through physical displacement. The resulting structural instability leads to membrane blebbing and OMV release. Roier et al. directly linked phospholipid accumulation in the outer membrane to vesiculation by showing that inactivation of the VacJ/Yrb ABC transport system in *Haemophilus influenzae* increases OMV production (24). This phospholipid-dependent mechanism appears evolutionarily conserved and is subject to precise genetic regulation, for example, through the ferric uptake regulator (Fur) system, illustrating how bacteria actively modulate OMV production in response to environmental cues.

### 2.4 Additional regulatory factors

Beyond the established models, emerging evidence indicates that various exogenous factors significantly modulate OMV biogenesis. Environmental stimuli such as temperature, ultraviolet radiation, and nutritional factors have been shown to regulate OMV production both quantitatively and qualitatively, affecting vesicle yield, size distribution, and molecular cargo composition (25, 26). This underscores that bacterial vesiculation is a dynamic response to extracellular conditions.

While the models described above provide valuable frameworks, none alone fully explains the complexity of OMV production across all contexts. The process is under intricate genetic control, with numerous genes implicated in its biosynthesis and regulation (summarized in **Table 1**). For example, utilizing a novel curvature-sensing fluorescent peptide (nFAAV5-NBD) in a genome-wide screen of *Shewanella vesiculosa*, Hiromu’s team identified several key genetic regulators of vesiculation, including putative dipeptidyl carboxypeptidase, glutamate synthase β-subunit, LapG protease, metallohydrolase, RNA polymerase sigma-54 factor, inactive transglutaminase, PepSY domain-containing protein, and Rhs-family protein (27). Importantly, elucidating the fundamental mechanisms of OMV biogenesis holds substantial practical value. A deeper mechanistic understanding is crucial for rationally optimizing production yields and cost-efficiency, which represents a major bottleneck in advancing therapeutic OMV applications.

**Table 1:** Genes involved in bacterial OMV biosynthesis and regulation.

Related genes	Reported bacteria	Functions	References
*OprF* (its homolog *OmpA*)	1.*Pseudomonas plecoglossicida* 2.*Pseudomonas aeruginosa*	1.OprF is a hydrophilic channel-forming protein contributing to OM permeability; 2.OprF is involved in adhesion to eukaryotic cells and in biofilm formation under anaerobic conditions; 3.OprF binds gamma interferon, which leads to the stimulation of the quorum sensing (QS) network and consequently to the production of two bacterial virulence factors, the lectin PA1 and the phenazine pyocyanin; 4. OprF has been suggested to be a bacterial sensor, involved in the perception of the host immune state; 5. Removal of the entire OprF protein results in a hypervesiculation phenotype.	(107–109)
*OmpA*	Gram-negative bacteria like *Escherichia coli* (*E. coli*), *Salmonella* and *Vibrio cholerae* (*V. cholerae*)	1. Gram-negative bacteria outer membrane β-barrels protein family; 2. An increased vesiculation due to deletion or truncation of *OmpA*, representing the OMP P5 homologue and an abundant protein linking the OM and peptidoglycan layer.	(24, 110)
*pepP*	*E. coli*	1. *PepP* is a proline aminopeptidase gene; 2. Mutations in *pepP* results in hypovesiculating.	(34, 111)
*ypjA*	*E. coli*	1. YpjA is a hypothetical Omp; 2. Mutations in *ypjA* results in hypovesiculating.	(112)
*bolA*	*E. coli*	1. BolA is thought to regulate morphology changes as well as increased resistance to antibiotics and detergents in stationary phase and under conditions of stress; 2. Mutations in *bolA* results in hypovesiculating.	(113)
*dsbA*	*E. coli*	1. DsbA is a disulfide oxidoreductase which aids in periplasmic protein folding by inducing disulfide bonds; 2. Mutation of *dsbA* results in hypovesiculating and deletion mutant of the factor responsible for dsbA recycling, dsbB act as a hypovesiculator.	(114, 115)
Tol-pal genes	*E. coli*, *Helicobacter Pylori*, *Salmonella Choleraesuis* and other Gram-negative bacteria	1. Tol-Pal system is a multiprotein complex of five envelope proteins, TolQ, TolR, TolA, TolB, and Pal; 2. The TolA, TolQ, and TolR inner membrane proteins are associated via their transmembrane segments; 3. The TolB periplasmic protein interacts with the Pal outer membrane lipoprotein; 4. Pal, a peptidoglycan-associated lipoprotein, is an outer membrane-anchored protein; 5. The tol-pal cluster encodes two additional proteins: cytoplasmic Orf1 and periplasmic Orf2; 6. The system plays numerous biologic functions in Gram-negative bacteria, including cell morphology, sensitivity to bile salts, and bacterial virulence; 7. Inactivation of the tol–pal genes negatively impact the outer membrane integrity, resulting in increased formation of OMVs.	(116–119)
*yfgL*	*E. coli*	1.*YfgL* encodes a lipoprotein involved in the syntheses and/or degradation of peptidoglycan (PG); 2. Its role in vesicle budding is proposed to be the result of an increase in PG production, or the down-regulation of lytic transglycosylases that leads to a loss of turgor pressure on the OM; 3. Deletion of the *yfgL* gene resulted in a decreased release of OMVs.	(120)
*VacJ/YrbE*	Gram-negative bacteria like *Haemophilus influenzae* and *V. cholerae*	1. VacJ/Yrb ABC (ATP-binding cassette) transport system is a proposed phospholipid transporter; 2. MlaA/mlaE in *E. coli* is involved in phospholipid accumulation; 3. Deletion or repression of *VacJ/Yrb* (*mlaA/mlaE*) increases OMV production.	(24, 121)
*mreB*	*Haemophilus influenzae*	1. MreB is a rod shape-determining protein; 2. *MreB* deletion mutant will promote the release of OMVs.	(24)
*dsbC*	*Haemophilus influenzae*	1. Thiol-disulfide interchange protein; 2. The absence of *dsbC* promote the formation of OMV by affecting outer membrane stability or stress response pathways.	(24)
*gmhA*	*Haemophilus influenzae*	1. Phosphoheptose isomerase; 2. The absence of *gmhA* related to outer membrane stability, thereby promoting the release of OMVs.	(24)
*rseA*	*E. coli*	1. Transmembrane anti-sigma factor; 2. *RseA* gene deletion increases OMVs production.	(122, 123)
*HylF*	*E. coli* and *Salmonella enterica*	1. Hemolysin F (HlyF) lacks hemolytic activity acting as a cytoplasmic enzyme, and capable of delivering a bona fide hemolysin ClyA; 2. Triggering the formation of OMVs.	(124)
*CprA*	*Pseudomonas aeruginosa*	1. CprA is an a short-chain dehydrogenase/reductase (SDR) contributing to resistance against colistin and antimicrobial peptides; 2. CprA is a functional ortholog of HlyF induce OMVs formation (These SDRs also induce the production of OMVs which block autophagic flux).	(125)
*PagC*	*Salmonella enterica*	1. Salmonella outer membrane protein PagC is a major constituent of Salmonella membrane vesicles; 2. PagC is controlled in a sigmaS-dependent manner; 3. Overexpression of *PagC* in bacteria could accelerate the generation of OMVs.	(126)
*OmpX*	Gram-negative bacteria	1. OmpX belongs to a family of onserved proteins, including PagC from S. enterica serovar Typhimurium; 2. OmpX is an eight β-stranded transmembrane barrel protein that is integrated into the outer membrane; 3. Overexpression of *OmpX* gene in bacteria could accelerate the generation of OMVs.	(126, 127)
*degP*	*E. coli*	1. The *degP* gene encodes a periplasmic protease/chaperone (sigmaE regulon) that manages unfolded and misfolded periplasmic proteins; 2. Disruption of the DegP protease or associated stress-response components (DegS/RseA) enhances OMV biogenesis, likely through periplasmic misfolded protein accumulation.	(121, 128)
*MicA*	*E.coli* and conserved among enterobacteria	1. *MicA* is a small non-coding RNA gene; 2. MicA is an efficient inducer of OMVs by regulating OmpA; 3. MicA-derived OMVs are OmpC-enriched.	(129)
*VrrA*	*V. cholerae*	1. *VrrA* is a small non-coding RNA gene; 2. VrrA is a homolog of MicA; 3. Induction of sRNA VrrA increased OMV production in a manner comparable with the loss of OmpA; 4. sRNA VrrA could be used for large-scale production of OMVs.	(110)
*NlpI*	*E.coli*	1. NlpI is an outer-membrane lipoprotein associated with cell division; 2. Increased PG dynamics in the *nlpI* mutant, linked to reduced Lpp-OM cross-linking, drives hypervesiculation; 3. Spr is identified as a PG DD-endopeptidase; 4. penicillin-binding protein (PBP) 7 is a murein DD-endopeptidase; 5. NlpI indirectly modulate OMVs production through negatively regulates either Spr activity during stationary phase or PBP4 during log phase.	(130)

## 3 Main composition of OMVs

Gram-negative bacteria naturally release OMVs, which encapsulate approximately 0.2%-0.5% of their OM and periplasmic components to form sophisticated, biomolecule-rich nanostructures (28). Structurally, OMVs maintain a characteristic bilayer architecture composed primarily of phospholipids, fatty acids, and cholesterol. This lipid-based framework provides both structural integrity and a vehicle for intercellular communication and virulence factor delivery. Unlike mammalian extracellular vesicles, OMVs contain unique bacterial components including LPS and PG (29). The vesicular cargo is composed of membrane-derived signaling lipids, genetic material, protein effectors including enzymes and toxins, as well as pathogen-associated molecular patterns. This assemblage of components collectively empowers OMVs to mediate a wide array of essential functions. Collectively, this multifaceted composition underpins the remarkable functional versatility of OMVs in microbial physiology and host-pathogen interactions, establishing them as pivotal mediators in bacterial survival and pathogenesis.

### 3.1 LPS

LPS is a complex molecule localized in the outer leaflet of the Gram-negative bacteria OM. Historically, LPS was first described as “endotoxin” by Pfeiffer, who characterized it as a highly toxic component of *Vibrio cholera* (30). Consequently, LPS constitutes the primary virulence factor in OMVs derived from Gram-negative bacteria. Notably, LPS exhibits significantly enhanced toxicity when associated with OMVs compared to its free form (31). This enhanced pathogenicity is exemplified by the unique capacity of OMVs to transport LPS across the blood-brain barrier, inducing inflammation in both the peripheral and central nervous system - a feat unattainable by purified LPS alone (32). The potent inflammatory potential of OMV-associated LPS is further demonstrated by observations that intranasal administration of *P. aeruginosa* OMVs elicits stronger pulmonary inflammation than equivalent doses of purified LPS, underscoring OMVs’ efficient delivery capability (33). At the molecular level, LPS recognition occurs via TLR4-mediated signaling: the LPS-TLR4 complex recruits TIRAP and MyD88 to activate IRAK and TRAF6, ultimately stimulating NF-κB and MAPK pathways (13). Notably, OMV-mediated delivery can bypass this surface recognition, facilitating LPS entry into the host cell cytosol where it triggers caspase-11-dependent immune responses (34). The 3-O-desacyl-4’ monophosphoryl lipid A (MPL, Corixa), derived from LPS of *Salmonella Minnesota* R595, is an approved adjuvant in human vaccines (35). These findings collectively emphasize the dual nature of OMV-associated LPS: while its endotoxin activity poses a challenge for therapeutic safety, its potent immunostimulatory properties, when strategically modulated through bioengineering, present significant opportunities for novel vaccine design.

### 3.2 PG

PG is the main component of the prokaryotic cell wall. It is a multi-layered, network-like macromolecular formed by the polymerization of N-acetylglucosamine (GlcNAc), N-acetylmuramic (MurNAc), and four to five amino acid short peptides. Notably, the human receptors NOD1 exhibits remarkable specificity for the GlcNAc-MurNAc tripeptide motif containing diaminopimelate (GM-TriDAP), a structural signature predominantly associated with Gram-negative bacterial PG (36). Upon binding this ligand, NOD1 initiates a signaling cascade via its caspase activation and recruitment domain (CARD). This involves recruiting receptor-interacting protein 2 (RIP2) to activate both NF-κB and MAPK pathways and ultimately driving the production of proinflammatory cytokines such as IL-8 (37). Intriguingly, while extracellular administration of synthetic or natural NOD1 agonists fails to activate NOD1 signaling in non-phagocytic epithelial cells, Kaparakis et al. demonstrated that Gram-negative bacterial PG delivered via OMVs can efficiently enter these cells through lipid raft-mediated endocytosis, thereby triggering robust NOD1-dependent immune responses (38). This OMV-mediated delivery mechanism provides critical insights into how Gram-negative bacteria may promote inflammatory pathology during infection by subverting normal immune recognition pathways.

### 3.3 Enzymes and toxins

Proteins constitute a functionally diverse and critical component of OMVs, encompassing virulence factors, antibiotic resistance determinants, and host interaction mediators. These vesicle-associated proteins facilitate intercellular communication and regulation, thereby enhancing bacterial survival and dissemination capabilities.

A key facet of OMV functionality is their role as specialized vehicles for delivering bacterial toxins and enzymes into eukaryotic cells. Enterotoxigenic *Escherichia coli* exemplifies this mechanism by packaging heat-labile enterotoxin (LT) within composite vesicular structures derived from both OM and periplasmic components (39). Similarly, *P. aeruginosa* employs OMVs as an alternative secretion pathway for multiple virulence factors, including phospholipase C, protease, hemolysin, and alkaline phosphatase - enzymes that collectively contribute to the pathogenesis of *Pseudomonas* infections (40). The periodontal pathogen *Aggregatibacter actinomycetemcomitans* employs a related strategy by displaying leukotoxin on OMV surfaces, facilitating its involvement in both localized aggressive periodontitis and systemic disease (41). *Bacteroides thetaiotaomicron* targets host immune cells via OMVs harboring bacterial sulfatase activity, demonstrating how OMVs-associated enzymes can promote inflammatory immune stimulation in genetically susceptible hosts (42). It is worth noting that clyA, a pore-forming cytotoxin expressed by Enterobacteriaceae, is also secreted via OMVs, a finding with important implications for biotechnology applications (43). The systematic characterization of OMV-associated proteins remains a crucial research direction, offering insights into fundamental microbial pathogenesis and revealing potential avenues for therapeutic applications.

### 3.4 Nucleic acids

OMVs serve as natural carriers for various nucleic acid species, including both DNA and RNA, which play crucial roles in bacterial gene regulation and intercellular genetic exchange. The incorporation of DNA into OMVs may occur through multiple pathways, ranging from spontaneous encapsulation during cell lysis events to selective packaging mechanisms (44). Compelling evidence for non-random DNA packaging comes from Bitto et al., who demonstrated preferential enrichment of specific chromosomal regions, encoding virulence factors, stress response elements, antibiotic resistance determinants, and metabolic enzymes, within *P. aeruginosa* OMVs. Their findings revealed a distinctive spatial organization, with most DNA associated with the vesicle surface and smaller quantities contained internally (45). The role of OMVs in mediating HGT is well-documented across multiple bacterial species. Dorward et al. established that *Neisseria gonorrhoeae* utilizes OMVs as vectors for intercellular plasmid transfer under physiological conditions (46). This phenomenon has been corroborated by numerous studies demonstrating OMV-facilitated dissemination of genetic material encoding diverse functions, including metabolic pathways, virulence traits, and antibiotic resistance markers (47).

In addition to DNA, various RNA species are packaged into OMVs, and their contributions to host-microbe interactions have been confirmed in several studies (48). The majority of OMV-associated RNAs are small RNAs (sRNAs), including microRNAs (miRNAs), and miRNA-sized sRNAs (msRNAs) (48, 49). A striking example is provided by *Porphyromonas gingivalis*, whose OMVs deliver sRNA-23392 to promote oral squamous cell carcinoma progression by inhibiting desmocollin-2 expression (50). Despite these advances, the molecular mechanisms governing the selective sorting of RNA into OMVs remain poorly understood, representing a critical knowledge gap in the field.

## 4 Functions of OMVs

OMVs serve as multifunctional biological nanoparticles pivotal to bacterial physiology and host-microbe interactions. Bearing diverse cargo, these sophisticated nanostructures mediate both interbacterial communication and cross-kingdom signaling. As the sole known mechanism for delivering hydrophobic compounds to host cells, OMVs have been formally classified as type 0 secretion systems (51). Their functional spectrum encompasses nutrient acquisition, environmental adaptation, and antibiotic resistance dissemination through HGT. In pathogenic contexts, OMVs act as precision delivery vehicles for virulence factors, facilitating host cell invasion while simultaneously modulating immune responses. They also contribute significantly to biofilm architecture and stability, enhancing bacterial community resilience. This remarkable functional versatility derives from their composition dynamically adapts to environmental cues, enabling homeostasis and ecological responsiveness. The capacity of OMVs to participate in such diverse biological processes underscores their fundamental role in microbial ecology and pathogenesis.

### 4.1 Bacterial defense mechanisms

OMV biogenesis represents an evolutionarily conserved defense strategy employed by bacteria to cope with environmental stressors. Under nutrient limitation, antibiotic pressure, or other extreme challenges, bacteria actively modulate OMV production to enhance survival through multiple protective mechanisms (52). These include neutralizing membrane-targeted threats, scavenging harmful extracellular compounds, and dynamically remodeling vesicular protein composition (53). *Vibrio fischeri* upregulates its major OMP OmpU under acidic pH encountered during host colonization. This pH-dependent protein restructuring significantly alters OMV cargo composition to facilitate host-symbiont interactions (54). *Pseudomonas putida* rapidly releases OMVs upon stress exposure, increasing cell surface hydrophobicity to promote biofilm formation and persistence (55). OMVs from enterotoxigenic *Escherichia coli* (*E. coli*) can quickly and irreversibly bind T4 bacteriophage, allowing parental bacteria to effectively evade OM-target attack (56).

OMVs function as a critical defense mechanism against antibiotics by shielding bacteria from antibiotic-induced damage and by acting as export vehicles for antibiotic-degrading enzymes. For instance, the Antarctic bacterium *Pseudomonas syringae* produces OMVs that mimic parent cell, effectively neutralizing membrane-active antibiotics (57). Similarly, *E. coli* secretes the metallo-β-lactamase NDM-1 via OMVs. These vesicles act as extracellular reservoirs of the enzyme to degrade β-lactam antibiotics in the environment and protect neighboring bacterial populations (58). Additionally, OMVs production increases when bacteria are briefly exposed to low doses of antibiotics, as OMVs can reduce antibiotic concentration by interacting with antibiotics. Specifically, sublethal doses of β-lactams antibiotics can induce hypervesiculation in *Salmonella enterica* sv. Typhi. The newly generated OMVs exhibit high affinity for polymyxin B, sequestering it and thereby enhancing bacterial survival against this agent (59). These examples underscore how OMV production serves as a versatile, rapid-response defense system that enhances bacterial survival across varied ecological niches and stress conditions.

### 4.2 Communication within bacterial populations

OMVs are vital for intercellular signaling, carrying various signaling molecules to coordinate group behavior, a process referred to as quorum sensing (QS). The differential content of their structural components and the selective packaging of biomolecules indicate that OMVs may function as “communicasomes,” mediating communication not only within the bacterial communities but also across different domains of life.

OMV enables the transfer of information between bacteria, which affects biological processes such as population behavior, growth, and metabolism. A prime example is *P. aeruginosa*, in which the hydrophobic signal PQS cannot diffuse freely across bacterial membranes and therefore relies on OMV packaging for transport. Studies indicate that more than 86% of the PQS produced by *P. aeruginosa* is packaged into OMVs (60). Removal of these OMVs from the bacterial community disrupts intercellular communication and suppresses PQS-controlled group behaviors. Similarly, the long-chain N-acyl-homoserine lactones, such as N-hexadecanoyl-L-homoserine lactone used by *Paracoccus denitrificans*, are disseminated via OMVs. These vesicles fuse selectively with recipient cells to facilitate signal delivery (61). The marine pathogen *Vibrio harveyi* packages the hydrophobic QS molecule CAI-1 into OMVs, stabilizing it in aqueous environments and extending its signaling range (62).

Beyond signaling, OMVs mediate bacterial antagonism by delivering antimicrobial cargo to eliminate competitors. For instance, *P. aeruginosa* secretes peptidoglycan hydrolases via OMVs to degrade rival bacteria (63). *Myxococcus xanthus* produces OMVs laden with proteases, phosphatases, and hydrolases that lyse *E. coli* cells upon membrane fusion (64). These findings align with the concept of OMVs as predatory tools in microbial warfare (65). Moreover, OMVs have also been identified as mediators of HGT in interspecies communication. Plasmids packaged within OMVs serve as the basis for HGT, thereby facilitating the spread of beneficial mutations. For example, OMVs from *Acinetobacter baylyi* (*A. baylyi*) transfer DNA to *E. coli* and other *A. baylyi* cells (66). Although interspecific exchange of OMVs is common in microbial communities, the mechanisms underlying how bacteria recognize and take up OMVs from different species remain elusive.

### 4.3 Communication between host and bacteria

OMVs are critical mediators of host-bacteria interactions, primarily through several key mechanisms, including host cell invasion, targeted delivery of virulence factors, and immunomodulation. As discussed above, OMVs carry numerous virulence factors, such as toxins, degrative enzymes, and adhesins. Crucially, compared to their free forms, OMV-associated virulence factors exhibit enhanced efficacy due to increased local concentration and protection from degradation. Active Cholera toxin can be delivered to intestinal epithelial cells by OMVs avoiding degradation by intestinal proteases (67). Certain periodontal pathogen-derived OMVs carry sRNAs that suppress cytokine expression in Jurkat T cells, highlighting their capacity for immune modulation (68).

Components like LPS and flagellin act as pathogen-associated molecular patterns, directly modulating host immune pathways. *Jang et al.*, found that the intraperitoneal administration of OMVs triggered significant increases in TNF-α and IL-6 levels in both serum and bronchoalveolar lavage fluid (69). Additionally, OMVs are small enough to even cross the blood-brain barrier (BBB) and induce an immune response. Studies have shown that extracellular RNAs in periodontopathogenic OMVs can successfully cross the BBB and prompt TNF-a production in murine brain (70). Mature OMVs are capable of adhering to the host cell membrane following their release and detachment, and subsequently enter the cell via endocytosis or membrane fusion to release their cargo at specific locations (71). This process can be influenced by the factors such as the size of OMVs themselves, LPS types, and glycoproteins (72, 73). The protective membrane structure of OMVs preserve the stability of these molecules, enabling them to modulate interactions and responses between bacteria and cells via long-distance transmission. Leveraging this property, OMVs can be harnessed for the treatment of intracellular infections, and antimicrobial-loaded OMVs enter host cells infected by pathogenic bacteria through endocytic or membrane fusion pathways.

OMV can act as potent mediators to enhance vaccine and drug delivery by efficiently accessing the lymphatic system and stimulating immune responses. This efficacy stems from their ability to fuse easily with target cell membranes, their long circulation time in the bloodstream, as well as their natural capacity for loading biomolecules (74). *Yersinia enterocolitica* OMVs elicit robust humoral and mucosal immune responses in murine models (75). Engineered OMVs have demonstrated remarkable antitumor effects by inducing IFN-γ-dependent tumor regression, enhancing antitumor cytokines production, and displaying tumor antigens to stimulate antigen-specific immunity (76, 77). These strategies effectively inhibit tumor growth and metastasis while establishing long-term immunological memory. The versatility of OMVs across infectious disease and oncology underscores their potential as next-generation biotherapeutic platforms.

## 5 Biological modification and application of OMVs

Recent years have witnessed significant progress in the biological modification of OMVs, revolutionizing their potential in biomedical and biotechnological applications. Current strategies for engineering OMVs primarily focus on three key objectives: attenuating virulence factors to enhance safety, optimizing production yields for scalable manufacturing, and functionally enhancing vesicles to maximize therapeutic efficacy. Genetic engineering has emerged as a powerful tool for precisely tailoring OMV properties through the targeted manipulation of bacterial genes that regulate vesicle biogenesis and cargo sorting. By engineering the host bacterial strain, researchers can systematically reprogram OMV characteristics while maintaining their inherent biological functions. Concurrently, the integration of synthetic nanomaterials with natural OMV components has given rise to a new generation of biohybrid nanostructures.

### 5.1 Licensed and clinical-stage OMV vaccines

OMV-based vaccines (VA-MENGOC-BC, MenBvac, MeNZB, and 4CMenB) have been employed worldwide in response to epidemic meningococcal disease outbreaks caused by *Neisseria meningitidis*. Meanwhile, several OMV-based candidates have advanced into clinical development, targeting a range of pathogens including bacteria and viruses.

Beyond licensed meningococcal vaccines, modified meningococcal OMVs have demonstrated versatility as platform technology in clinical development. A clinical trial in Brazil (MenB-Bio vaccine) evaluated a tailored OMV-based vaccine containing detergent-treated OMVs and detoxified lipooligosaccharide (dLOS) derived from two prevalent local MenB strains. The Phase II/III study demonstrates the clinical translation of modified, strain-specific OMVs designed to address regional meningococcal epidemiology (78). Genetically engineered meningococcal OMVs with constitutive expression of FetA (MenPF-1) completed Phase I clinical trials, showing enhanced bactericidal antibody responses beyond PorA-mediated immunity (79). The Walter Reed Army Institute of Research (WRAIR) has conducted pioneering clinical trials using genetically engineered native OMVs (nOMVs) derived from meningococci without exposure to detergent or denaturing agents. Early Phase I trials evaluated intranasal administration of nOMVs derived from capsule-negative strain 9162, demonstrating safety and induction of cross-reactive bactericidal antibodies despite the presence of LPS (80, 81). Subsequently, WRAIR developed intramuscular nOMV vaccines using strains with genetic modifications to enhance safety and immunogenicity. A phase 1 trial assessed a candidate prepared from a *lpxL2*(−) *synX*(−) mutant of strain 44/76 with stabilized opcA expression. This vaccine showed an acceptable safety profile and induced bactericidal antibodies, supporting its potential to provide protection against group B meningococcus (82). Furthermore, meningococcal OMVs are being explored as delivery platforms for heterologous antigens from non-bacterial pathogens. For instance, the SARS-CoV-2 Spike protein displayed on *Neisseria meningitidis* OMVs (Avacc 10), developed by IntraVacc B.V., has received authorization to enter Phase I clinical trials (NCT05604690).

OMV vaccines against *Neisseria gonorrhoeae* have garnered significant clinical interest in recent years. The 4CMenB and MeNZB vaccines have shown a protective effect against gonorrhoea, reducing acquisition by 30-40% (83). Additionally, a gonococcal generalized modules for membrane antigens (GMMA) vaccine has completed preliminary preclinical studies and is reported in the literature to be advancing toward clinical evaluation (84). GMMA are genetically engineered OMVs designed to achieve hypervesiculation and tailored membrane composition. This platform features a simple manufacturing process, leading to affordable vaccines (85). The first clinically tested GMMA vaccine, *Shigella sonnei* 1790GAHB, demonstrated safety and immunogenicity in phase I/II trials, including a human challenge model, though it failed to confer protection, likely due to insufficient O-polysaccharide dosing (86, 87). This finding prompted the development of a next-generation version with tenfold higher antigen content. The optimized GMMA has now been formulated into a tetravalent vaccine combining *Shigella flexneri* serotypes 1b, 2a, and 3a, which is currently undergoing phase I/II clinical evaluation (NCT05073003). A Phase I trial (SALVO study, ISRCTN51750695) evaluated the safety and immunogenicity of the bivalent invasive non-typhoidal *Salmonellae* (iNTS)-GMMA vaccine in healthy adults. The vaccine was well-tolerated with no serious adverse events and successfully induced persistent functional antibody responses. Despite the extensive reporting of OMV-based bacterial vaccine candidates in the scientific literature, most remain in the preclinical stage of development.

### 5.2 Strategies for lipid A-targeted attenuation of OMVs

The lipid A moiety of LPS constitutes the predominant and most extensively studied virulence factor in OMVs, making it the primary target for detoxification. Lipid A consists of a conserved (β1→6)-linked glucosamine disaccharide backbone, typically phosphorylated at the 1 and 4’ positions and acylated at the 2 and 3 positions of each sugar unit (88). Controlled modulation of lipid A architecture can balance immunogenicity with safety. Its immunostimulatory capacity critically depends on the acylation pattern, and hexa-acylated forms exhibit maximal cytokine-inducing activity via TLR4 activation, whereas tetra-acylated variants often act as TLR4 antagonists (89). Other structural features, including phosphate group number and acyl chain length, further modulate lipid A’s inflammatory potential (88). In *E. coli*, the Raetz pathway orchestrates lipid A biosynthesis through nine conserved enzymatic steps mediated by LpxA-LpxD, LpxH, LpxK, LpxM, and WaaA (90).

It is generally consensus that deleting late acyltransferases attenuates pathogens by leading to underacylated, less toxic lipid A species (91). LpxM and LpxL belongs to the lauryl/myristyl acyltransferases family involved in lipid A biosynthesis, and inactivation of either enzyme generates penta- or tetra-acylated lipid A species (92). In the *Shigella flexneri* 2a human challenge strain, deletion of the late acyltransferases *msbB1* and *msbB2* genes results in underacylated lipid A production (91). The 4’-phosphatase LpxF from *Francisella tularensis* dephosphorylates lipid A at the 4’ position in an *E. coli* LpxM mutant, and it does not act on wild-type hexa-acylated lipid A (93). LpxE from *Francisella novicida* selectively removes the 1-phosphate group of lipid A (94). Additionally, the palmitoyl transferase PagP in *E. coli* and *Salmonella* adds a secondary palmitoyl chain (C16) at the 2-position of lipid A, and such palmitoylated LPS exhibits attenuated signaling through the TLR4/MD-2 complex (95). In *Salmonella enterica*, Pag L hydrolyzes the ester bond at the 3-position of lipid A, thereby modulating lipid A recognition by the TLR4/MD-2 complex (96).

While the aforementioned lipid A engineering strategies provide powerful tools for endotoxin attenuation, translating these modifications into clinically viable OMV therapeutics requires navigating complex regulatory frameworks for endotoxin management. Regulatory agencies impose strict endotoxin limits for parenteral biologics, with FDA guidelines typically requiring ≤5 EU/kg/hour for systemic administration. However, these thresholds, established for conventional pharmaceuticals, may not appropriately reflect the risk-benefit profile of OMV-based immunotherapeutics where controlled TLR4 activation is often desirable for adjuvant effects (97). Application-specific endotoxin specifications are emerging: OMV vaccines may tolerate higher endotoxin levels to leverage immunostimulation, while OMV drug carriers require maximal detoxification. Critical regulatory considerations include establishing validated endotoxin quantification methods suitable for vesicular formulations, as standard limulus amebocyte lysate/tachypleus amebocyte lysate (LAL/TAL) assays may underestimate or overestimate activity in complex lipid matrices. Demonstrating batch-to-batch consistency in lipid A structure and TLR4 activity through orthogonal analytical methods such as mass spectrometry and cell-based potency assays is essential for regulatory approval. Correlating *in vitro* endotoxin measurements with *in vivo* pyrogenicity and safety through rabbit pyrogen tests or monocyte activation tests remains a regulatory requirement, though these methods have limitations for complex biological products. Defining acceptable endotoxin ranges that balance safety with therapeutic efficacy for each OMV application requires extensive preclinical dose-ranging studies with comprehensive toxicity assessment. The establishment of OMV-specific guidance, currently absent from key regulatory agencies, would facilitate standardized approaches to endotoxin risk assessment and management.

### 5.3 Cutting-edge technologies for functional enhancement of OMVs

Due to their nanoscale dimensions, OMVs possess ideal biophysical properties for immune system engagement. They are readily internalized by antigen-presenting cells, efficiently trafficked through lymphatic vessels, and demonstrate superior capabilities for antigen delivery, thereby potently stimulating both humoral and cellular immune responses. Recent advances in bioengineering have further expanded this therapeutic potential. The convergence of OMV biology with cutting-edge nanotechnology and genetic engineering is now opening new frontiers in biomedical research, creating unprecedented opportunities for the development of next-generation vaccines and immunotherapies.

#### 5.3.1 Cell membrane-coated nanoplatforms for advanced biomedical application

Cell membrane coating technology has emerged as a powerful strategy in biomedical research, particularly for vaccine development and targeted therapeutics. Coating synthetic nanoparticles with OMVs preserves the complex biological characteristics of bacteria and mimics the natural process of antigen presentation to the immune system. OMVs derived from *Bordetella bronchiseptica* were conjugated with PEGylated nano-Rehmannia glutinosa polysaccharide to synthesize a nanovaccine, which exhibits enhanced protective immunity against bacterial infection by modulating T-cell receptor signaling and Th1/Th2/Th17 differentiation (98). OMVs from *E. coli* Nissle 1917 served as nanoreactors to fabricate biomimetic copper sulfide nanoparticles, exhibiting high photothermal conversation efficacy, excellent photostability, and significant tumor-targeting capability (99). The OMV-based camouflage of gold nanocages facilitated the targeted uptake of dexmedetomidine-loaded nanoparticles into M1-like senescent macrophages, leading to a marked increase in drug delivery efficiency (100). In summary, coating nanocarriers with OMVs enhances the immunostimulatory effects of free OMVs by improving their stability and targeting capability. These biomimetic systems combine the advantages of artificial nanoparticles with the biological functionality of OMVs. The OMV-coating strategy offers several key benefits. It can enhance stability of therapeutic payloads, improve targeting specificity through natural membrane proteins, and amplify immunostimulatory effects via pathogen-associated molecular patterns. Such hybrid platforms bridge the gap between synthetic and biological systems. However, the extent of these improvements depends on the selected nanoparticle core, underscoring the need for optimal nanocarrier design.

#### 5.3.2 OMVs as versatile drug delivery vehicles

The concept of utilizing OMVs for drug delivery was first proposed in the late 1970s and early 1980s. Since then, OMVs have emerged as promising nanocarriers capable of encapsulating drugs or bioactive molecules, enabling targeted delivery to specific tissues or cells while enhancing drug bioavailability and therapeutic efficacy. Therapeutic agents can be loaded into OMVs via either energy-dependent (active loading) or energy-independent (passive loading) techniques (101). A key advancement in OMV-based delivery involves genetically modifying to display functional proteins on their surface. Outer membrane protein A (OmpA) has been used to stably display phosphotriesterase (PTE) from *Brevundimonas diminuta* while preserving its enzymatic activity (102). Cytolysin A (clyA) has been engineered to display affiEGFR-GALA peptides on OMVs derived from an endotoxin-reduced *E. coli* W3110 strain. These engineered OMVs serve as potential carriers for cell-specific drug delivery, particularly against cancer cells, as they are capable of reaching the cytoplasm while escaping the endosome due to the incorporation of a fusogenic GALA peptide. Selecting an appropriate anchor protein is crucial for ensuring effective surface display of recombinant proteins (103). A summary of commonly used anchors is provided in **Table 2**.

**Table 2:** Anchor Proteins in OMVs with potential for modification and application.

Anchor proteins	Function/potential application	Reference
clyA	ClyA is a pore-forming cytotoxin of enterobacteria, acting as a membrane anchor, ensuring the efficient display of antigens on the OMV surface.	(131)
OmpA	1. Outer membrane protein A (aminoacids 1-131), which can be fused by heterologous proteins on OMV surface; 2. OmpA as a multifunctional virulence factor plays an important role in the biogenesis of OMVs, biofilm formation, and resistance to different types of antimicrobial drugs.	(132, 133)
Lpp-OmpA	1. Lpp-OmpA is a membrane anchor protein, which can be effectively exploited for the presentation of desired proteins on the OMV surface; 2. This anchor consists of the transmembrane domain (aminoacids 46-159) from OmpA as well as the signal peptide and the first 9 N-terminal amino acids of the *E. coli* lipoprotein (Lpp).	(134, 135)
Hbp	1. *E. coli* autotransporter Hemoglobin protease (Hbp); 2. Heterologous proteins can be fused directly to the C-terminal β-domain or to the N terminus of the passenger domain; 3. The mature Hbp passenger’s β-helical stem (~100 Å) serves as a scaffold for replaceable side domains (d1-d5), enabling β-domain mutation-controlled surface display of heterologous proteins; 4. The Hbp platform was demonstrated to be a versatile tool for the simultaneous display of multiple sizeable antigens at the surface of OMVs.	(136–138)
OmpF	1. Porins involved in diffusion of ions and molecules; 2. Recombinant OmpF protein from *E. coli* could stimulate strong immunoglobulin G (IgG) antibody responses and provided high protection against lethal infection in mice with the highly virulent *E. coli* strain; 3. OmpF is a prominent porin, form a closely interconnected network on the surface of *E. coli*; 4. Heterologous protein insert into OmpF, and the modification is stable over 30 passages of the recombinant bacteria on the DNA and protein level.	(139)
MipA	1. *E. coli* MltA-interacting protein (MipA); 2. MipA (aminoacids 1-140) can be used as a novel anchoring motif for efficient bacterial surface display especially for enzymes in the biotechnological and industrial fields; 3. MipA is identified in OMVs from Cronobacter spp, and MipA has the potential to be an anchor protein on OMVs.	(140, 141)
LamB	1. LamB is a trimeric OMP that functions in the transport of maltose and maltose polymers and also serves as the receptor for bacteriophage λ; 2. LamB is rich in beta-structure, contain additional targeting information that directs proper membrane insertion; 3. The LamB maltoporin has been used successfully to display foreign peptides on the cell surface of *E. coli* as LamB hybrids, which has the potential to be an anchor protein on OMVs; 4. More than 200 amino acids have been cloned into lamB, but usually less than 60 amino acids can be inserted into it without affecting its surface location.	(142, 143)
OmpC	1. OmpC and OmpA were previously identified as OMV proteins, and they are often used as marker proteins for OMVs; 2. OmpC has the potential to be an anchor protein on OMVs.	(144)
OmpW	OmpW is constantly and abundantly expressed on the outer membranes of *E. coli* cells and their OMVs; Fusing GFP expression with OmpW in *E. coli* cells enabled the packaging of GFP protein into OMVs.	(145)
FhuA	1. FhuA is a natural iron transporter located in the outer membrane of *E. coli*; 2. Target of several bacteriophages and also a target of bacteriocins; 3. It forms a b-barrel structure exposing 11 loops on the bacterial surface, of which the largest, loops 4 and 5, tolerate inserts of up to 200 amino acids in length without disturbing surface expression or sensitivity to phages T5 and U80 or colicin M; 4. FhuA to be a suitable platform for various applications in biocatalysis, material science and the construction of artificial metalloenzymes; 5. OMVs enriched in FhuA, which has the potential to be an anchor protein on OMVs.	(146, 147)
PelB peptide signal	1. Pectate lyase subunit (PelB); 2. A truncated PelB signal sequence without the recognition site of signal peptidase; 3. A popular leader sequence able to deliver target protein to the periplasmic space; 4. Export target protein in the lumen of OMVs.	(148, 149)

Therapeutic mRNA vaccines have advanced rapidly in recent years. Concurrently, OMVs have been proved to be a promising mRNA delivery platform through genetic engineering. For example, OMVs genetically decorated with RNA-binding and lysosomal escape proteins (OMV-LL) can deliver mRNA antigens, markedly inhibiting tumor progression in models of metastatic and subcutaneous colon cancer (104). The successful demonstration of OMV-based mRNA delivery heralds a significantly advance in next-generation vaccine and immunotherapies. Meanwhile, bioconjugation systems, such as the SpyCatcher-SpyTag enables covalent protein coupling to OMV surfaces, offering a modular and efficient approach for functionalization. Additionally, CRISPR-based systems have been leveraged to enhance the efficiency of targeting and packaging therapeutic molecules into OMVs (105). These technologies further expand the possibilities for customizable OMV engineering, allowing for multifunctional drug delivery and immune modulation.

## 6 Summary and outlook

### 6.1 Challenges in OMV-based application

In clinical applications, the approval of several OMV-based meningococcal vaccines for human use highlights their potential, with promising prospects in vaccine development, targeted drug delivery, and tumor therapy. However, the clinical implementation of OMVs faces multiple substantial barriers.

OMV production is inherently constrained by bacterial physiological factors including microenvironmental conditions, viability status, and growth phase, leading to low yields that require costly optimization through culture condition refinement or genetic engineering approaches. Purification methods involve isolation via techniques such as ultracentrifugation, filtration, or chromatography, and these processes may introduce impurities including free proteins that affect OMV purity and safety.

Beyond production yield and purity, achieving batch-to-batch consistency represents a fundamental manufacturing challenge for OMV therapeutics. The biological nature of OMV production introduces inherent variability in critical quality attributes including vesicle size distribution, protein and lipid composition, antigen loading efficiency, and immunogenic potency. Controlling this requires stringent management of fermentation parameters such as pH, temperature, dissolved oxygen, growth phase, and media composition. This control must operate within narrowly defined ranges following validated bioprocess protocols. Furthermore, implementation of Process Analytical Technology (PAT) for real-time monitoring and adaptive control of OMV production has emerged as a promising approach to reduce batch variability. Subsequently, comprehensive release testing must encompass physical characterization through dynamic light scattering for size and polydispersity index, nanoparticle tracking analysis for concentration, biochemical profiling including proteomics and lipidomics, endotoxin quantification, and functional potency assays. Despite these measures, the establishment of certified reference standards for comparative batch analysis remains a critical unmet need in the field.

Current manufacturing compliance with cGMP requirements presents additional challenges, as existing frameworks are designed for mammalian cell culture or chemical synthesis rather than bacterial fermentation-derived vesicles. The lack of industry-wide standardization protocols and OMV-specific regulatory guidance from major agencies including FDA, EMA, and NMPA currently impedes inter-laboratory reproducibility and necessitates case-by-case regulatory evaluation. Establishing distinct regulatory pathways for OMV therapeutics rather than forcing classification into existing vaccine or drug categories is essential for streamlining approval processes while ensuring appropriate safety oversight.

Comprehensive safety assessment is paramount for clinical translation of OMV-based therapeutics. The immunostimulatory properties that make OMVs attractive for vaccines and immunotherapy simultaneously pose immunotoxicity risks requiring careful evaluation. Acute concerns include cytokine release syndrome from excessive TLR activation, particularly with high-dose or repeated administration, necessitating establishment of maximum tolerated doses and monitoring of pro-inflammatory cytokines including IL-6, TNF-α, and IL-1β in preclinical studies. Systemic inflammatory response syndrome risk is elevated in immunocompromised patients or those with pre-existing inflammatory conditions, necessitating patient stratification strategies and potentially exclusion criteria in early-phase clinical trials. Additionally, potential autoimmune reactions may arise if OMV-displayed antigens exhibit molecular mimicry with host epitopes, a concern that requires comprehensive epitope mapping and immunological profiling during preclinical development. Hypersensitivity reactions upon repeated exposure warrant investigation through appropriate animal models that recapitulate chronic dosing regimens. Mitigation strategies include lipid A detoxification as discussed in Section 4.1, dose optimization through careful pharmacokinetic and pharmacodynamic studies, premedication protocols to dampen excessive inflammatory responses, and comprehensive immunotoxicity panels including cytokine profiling, complement activation assays, and immunophenotyping in preclinical safety assessments.

The long-term safety profile of repeated OMV administration, particularly relevant for chronic immunotherapy regimens, remains inadequately characterized. Critical knowledge gaps include potential for immune tolerance or exhaustion with chronic exposure that could diminish therapeutic efficacy over time. Risk of persistent inflammation or organ damage in tissues with high OMV accumulation requires histopathological examination in extended toxicology studies. The impact of OMV administration on microbiome composition and host-microbe homeostasis represents an emerging safety consideration, as disruption of commensal bacterial communities could have unintended metabolic and immunological consequences (106). Reproductive and developmental toxicity for long-term treatments must be evaluated through standard reproductive toxicology study designs. Genotoxicity concerns related to bacterial DNA fragments potentially carried by OMVs require assessment. Establishing these safety parameters requires rigorous long-term toxicology studies in relevant animal models with extended observation periods of at least six months, repeated-dose regimens that accurately mimic proposed clinical protocols, and comprehensive histopathological examination of all major organ systems. For approved OMV products, pharmacovigilance programs with long-term patient follow-up are essential to detect rare or delayed adverse events not apparent in limited clinical trials.

The clinical translation of OMVs necessitates coordinated advances in scalable PAT-controlled bioprocessing, harmonized analytical and release criteria, comprehensive and predictive safety science, and the development of a tailored regulatory science framework. It is still worth noting that the targeting ability of OMVs and their circulation time in the bloodstream will influence their efficacy in clinical applications. If OMVs are easily cleared from the bloodstream or exhibit poor targeting ability, their antimicrobial or antitumor efficacy will be compromised.

### 6.2 Outlook

Notably, many clinical adjuvants, such as MPL and CpG, originate from bacterial components, and bacterial-based cancer therapies further highlight bacteria as valuable source of immunostimulatory agents. In contrast to live bacteria, OMVs are non-replicating and thus inherently safer, yet they retain natural immunogenicity and can be systematically engineered. Rather than being mere bacterial byproducts, OMVs represent versatile immunotherapeutic platforms beyond single-purpose vaccines. By integrating rapidly evolving synthetic biology tools with growing insights in microbiology and immunology, it is becoming feasible to precisely tailor OMV composition, targeting, and functionality. The OMV-based platforms allow for systematic and independent modification of both internal and surface-displayed antigens due to their modular nature. This flexibility enables rapid responses to emerging infectious threats, such as COVID-19, and other unforeseen public health challenges.

The characteristics and clinical applications of different OMV-based vaccine types are summarized in **Table 3**. The successful clinical application of OMVs represents not an endpoint but the inception of a broader paradigm shift in biomedical nanotechnology. The path forward requires deepening fundamental insights into OMV biogenesis and host interaction dynamics, coupled with the establishment of robust, scalable manufacturing and tailored safety-assessment frameworks. By uniting these foundational advances with translational engineering, OMV-based platforms are positioned to fulfill their potential as a versatile and transformative tool in precision medicine.

**Table 3:** Strengths and limitations comparison of different OMV-based vaccine types.

OMV-based vaccine types	Features	Representative pathogens/disease models	The merits	The drawbacks	Current development stage	References
Native OMVs	Naturally released vesicles purified with detergents to reduce LPS content	*Neisseria meningitidis*	High immunogenicity; Relatively simple production	Detergent use can lead to loss of key antigens; Heterogeneous composition; Potential for autoimmunity	Licenced (VA-MENGOC-BC, MenBvac); Clinical trials	(78)
Genetically attenuated OMVs	Gene knockout/modification to produce detoxified lipid A; deletion of virulence factors	*Salmonella* spp.; *Shigella flexneri*	Improved safety profile; Retains self-adjuvanticity; Genetically defined process	May slightly reduce immunogenicity; Requires genetic engineering expertise	Clinical trials; Preclinical	(150, 151)
Antigen-display OMVs	Genetic fusion of heterologous antigens to anchor proteins	SARS-CoV-2; Tumor therapy (displayed tumor antigens)	Targeted immune responses; Multivalent antigen presentation; No need for antigen purification	Correct folding and display can be challenging; Genetic fusion may affect OMV biogenesis	Clinical trials phase I (Avacc 10); Preclinical	NCT05604690
Cargo-loaded OMVs	Loading of therapeutic molecules (mRNA, drugs, proteins) via electroporation, sonication, or co-incubation	Directing enzyme packing delivery; Tumor therapy delivery	Protects cargo from degradation; Enables combination therapy; Versatile payload capacity	Low loading efficiency; Concerns about stability and toxicity; Potential damage to OMV structure; Uncontrolled release	Preclinical;	(104)
Hybrid/Biomimetic OMVs	Fusion of OMVs with synthetic liposomes or coating onto synthetic nanoparticle cores	Tumor immunotherapy; Drug delivery for chronic bacterial infections	Tunable properties; Enhanced stability; Synergistic functionality	Complex manufacturing process may affect the membrane topology and biofunctions; Batch-to-batch variability	Preclinical	(100)

## References

[1] Chatterjee SN Das J . Electron microscopic observations on the excretion of cell-wall material by Vibrio cholerae. J Gen Microbiol. (1967) 49:1–11. doi: 10.1099/00221287-49-1-1, PMID: 4168882

[2] Namork E Brandtzaeg P . Fatal meningococcal septicaemia with “blebbing” meningococcus. Lancet. (2002) 360:1741. doi: 10.1016/S0140-6736(02)11721-1, PMID: 12480427

[3] Briaud P Carroll RK . Extracellular vesicle biogenesis and functions in gram-positive bacteria. Infect Immun. (2020) 88:e00433–20. doi: 10.1128/IAI.00433-20, PMID: 32989035 PMC7671900

[4] Liu H Zhang Q Wang S Weng W Jing Y Su J . Bacterial extracellular vesicles as bioactive nanocarriers for drug delivery: Advances and perspectives. Bioact Mater. (2022) 14:169–81. doi: 10.1016/j.bioactmat.2021.12.006, PMID: 35310361 PMC8892084

[5] Zhu Z Antenucci F Villumsen KR Bojesen AM . Bacterial outer membrane vesicles as a versatile tool in vaccine research and the fight against antimicrobial resistance. mBio. (2021) 12:e0170721. doi: 10.1128/mBio.01707-21, PMID: 34372691 PMC8406158

[6] Schwechheimer C Kulp A Kuehn MJ . Modulation of bacterial outer membrane vesicle production by envelope structure and content. BMC Microbiol. (2014) 21:324. doi: 10.1186/s12866-014-0324-1, PMID: 25528573 PMC4302634

[7] Orench-Rivera N Kuehn MJ . Environmentally controlled bacterial vesicle-mediated export. Cell Microbiol. (2016) 18:1525–36. doi: 10.1111/cmi.12676, PMID: 27673272 PMC5308445

[8] Noundou VL Levy A Modla S Yu Y Qu J Hanson TE . Chlorobaculum tepidum outer membrane vesicles may transport biogenic elemental sulfur. Appl Environ Microbiol. (2025) 91:e0101925. doi: 10.1128/aem.01019-25, PMID: 40530856 PMC12285230

[9] Johnston EL Zavan L Bitto NJ Petrovski S Hill AF Kaparakis-Liaskos M . Planktonic and biofilm-derived pseudomonas aeruginosa outer membrane vesicles facilitate horizontal gene transfer of plasmid DNA. Microbiol Spectr. (2023) 11:e0517922. doi: 10.1128/spectrum.05179-22, PMID: 36946779 PMC10100964

[10] Zhao M He M Lin X Wu K Yang F Chen X . Identification of outer membrane vesicles as a new vehicle mediating antibiotic resistance gene transfer in campylobacter. J Extracell Vesicles. (2025) 14:e70195. doi: 10.1002/jev2.70195, PMID: 41216876 PMC12603785

[11] Rueter C Bielaszewska M . Secretion and Delivery of Intestinal Pathogenic Escherichia coli Virulence Factors via Outer Membrane Vesicles. Front Cell Infect Microbiol. (2020) 10. doi: 10.3389/fcimb.2020.00091, PMID: 32211344 PMC7068151

[12] Juodeikis R Martins C Saalbach G Richardson J Koev T Baker DJ . Differential temporal release and lipoprotein loading in B. thetaiotaomicron bacterial extracellular vesicles. J Extracell Vesicles. (2024) 13:e12406. doi: 10.1002/jev2.12406, PMID: 38240185 PMC10797578

[13] Wang G Wang Y Ma F . Exploiting bacterial-origin immunostimulants for improved vaccination and immunotherapy: current insights and future directions. Cell Biosci. (2024) 14:24. doi: 10.1186/s13578-024-01207-7, PMID: 38368397 PMC10874560

[14] Holger DJ Lev KL Kebriaei R Morrisette T Shah R Alexander J . Bacteriophage-antibiotic combination therapy for multidrug-resistant Pseudomonas aeruginosa: *In vitro* synergy testing. J Appl Microbiol. (2022) 133:1636–49. doi: 10.1111/jam.15647, PMID: 35652690

[15] Li Q Ou Z Lin J Tang D He B Wu Y . Specific labeling of outer membrane vesicles with antibiotic-conjugated probe reveals early bacterial infections in blood. Nat Commun. (2025) 16:3535. doi: 10.1038/s41467-025-58676-8, PMID: 40229269 PMC11997070

[16] Li M Du C Guo N Teng Y Meng X Sun H . Composition design and medical application of liposomes. Eur J Med Chem. (2019) 164:640–53. doi: 10.1016/j.ejmech.2019.01.007, PMID: 30640028

[17] Premchandani T Umekar M Tatode A Taksande J Khan R Faizan M . Extracellular vesicles in host–pathogen interactions: roles of exosomes and bacterial outer membrane vesicles in immunity and microbial communication. Bacteria. (2025) 4:63. doi: 10.3390/bacteria4040063

[18] Luo Z Cheng X Feng B Fan D Liu X Xie R . Engineering versatile bacteria-derived outer membrane vesicles: an adaptable platform for advancing cancer immunotherapy. Advanced Sci. (2024) 11:e2400049. doi: 10.1002/advs.202400049, PMID: 38952055 PMC11434149

[19] Ho MY Liu S Xing B . Bacteria extracellular vesicle as nanopharmaceuticals for versatile biomedical potential. Nano Convergence. (2024) 11:1–30. doi: 10.1186/s40580-024-00434-5, PMID: 38990415 PMC11239649

[20] Saha S Lach SR Konovalova A . Homeostasis of the Gram-negative cell envelope. Curr Opin Mocrobiol. (2021) 61:99–106. doi: 10.1016/j.mib.2021.03.008, PMID: 33901778 PMC8577651

[21] Florez C Raab JE Cooke AC Schertzer JW . Membrane distribution of the pseudomonas quinolone signal modulates outer membrane vesicle production in pseudomonas aeruginosa. mBio. (2017) 8:e01034–17. doi: 10.1128/mBio.01034-17, PMID: 28790210 PMC5550756

[22] Schertzer JW Whiteley M . A bilayer-couple model of bacterial outer membrane vesicle biogenesis. mBio. (2012) 3:e00297–11. doi: 10.1128/mBio.00297-11, PMID: 22415005 PMC3312216

[23] Zhao X Wei Y Bu Y Ren X Dong Z . Review on bacterial outer membrane vesicles: structure, vesicle formation, separation and biotechnological applications. Microb Cell Factories. (2025) 24:1–14. doi: 10.1186/s12934-025-02653-9, PMID: 39833809 PMC11749425

[24] Roier S Zingl FG Cakar F Durakovic S Kohl P Eichmann TO . A novel mechanism for the biogenesis of outer membrane vesicles in Gram-negative bacteria. Nat Commun. (2016) 7:10515. doi: 10.1038/ncomms10515, PMID: 26806181 PMC4737802

[25] Schwechheimer C Kuehn MJ . Outer-membrane vesicles from Gram-negative bacteria: biogenesis and functions. Nat Rev Microbiol. (2015) 13:605–19. doi: 10.1038/nrmicro3525, PMID: 26373371 PMC5308417

[26] Taheri N Fällman M Wai SN Fahlgren A . Accumulation of virulence-associated proteins in Campylobacter jejuni Outer Membrane Vesicles at human body temperature. J Proteom. (2019) 195:33–40. doi: 10.1016/j.jprot.2019.01.005, PMID: 30641234

[27] Inoue H Kawano K Kawamoto J Ogawa T Kurihara T . Rapid screening and identification of genes involved in bacterial extracellular membrane vesicle production using a curvature-sensing peptide. J Bacteriol. (2025) 207. doi: 10.1128/jb.00497-24, PMID: 40183544 PMC12096838

[28] Kuehn MJ Kesty NC . Bacterial outer membrane vesicles and the host-pathogen interaction. Gene Dev. (2005) 19:2645–55. doi: 10.1101/gad.1299905, PMID: 16291643

[29] Toyofuku M Nomura N Eberl L . Types and origins of bacterial membrane vesicles. Nat Rev Microbiol. (2019) 17:13–24. doi: 10.1038/s41579-018-0112-2, PMID: 30397270

[30] Pfeiffer R . Untersuchengen über das Choleragift. Med Microbiol Immunol. (1892) 11:393–412. doi: 10.1007/BF02284303

[31] Chen SL Lei Q Zou XH Ma DD . The role and mechanisms of gram-negative bacterial outer membrane vesicles in inflammatory diseases. Front Immunol. (2023) 14. doi: 10.3389/fimmu.2023.1157813, PMID: 37398647 PMC10313905

[32] Lyte M . Microbial endocrinology in the microbiome-gut-brain axis: how bacterial production and utilization of neurochemicals influence behavior. PloS Pathog. (2013) 9:e1003726. doi: 10.1371/journal.ppat.1003726, PMID: 24244158 PMC3828163

[33] Park K-S Lee J Jang SC Kim SR Jang MH Lötvall J . Pulmonary inflammation induced by bacteria-free outer membrane vesicles from Pseudomonas aeruginosa. Am J Resp Cell Mol. (2013) 49:637–45. doi: 10.1165/rcmb.2012-0370OC, PMID: 23713467

[34] Vanaja SK Russo AJ Behl B Banerjee I Yankova M Deshmukh SD . Bacterial outer membrane vesicles mediate cytosolic localization of LPS and caspase-11 activation. Cell. (2016) 165:1106–19. doi: 10.1016/j.cell.2016.04.015, PMID: 27156449 PMC4874922

[35] Didierlaurent AM Morel S Lockman L Giannini SL Bisteau M Carlsen H . AS04, an aluminum salt- and TLR4 agonist-based adjuvant system, induces a transient localized innate immune response leading to enhanced adaptive immunity. J Immunol. (2009) 183:6186–97. doi: 10.4049/jimmunol.0901474, PMID: 19864596

[36] Magalhaes JG Philpott DJ Nahori M-A Jéhanno M Fritz J Bourhis LL . Murine Nod1 but not its human orthologue mediates innate immune detection of tracheal cytotoxin. EMBO Rep. (2005) 6:1201–7. doi: 10.1038/sj.embor.7400552, PMID: 16211083 PMC1369207

[37] Irving AT Mimuro H Kufer TA Lo C Wheeler R Turner LJ . The immune receptor NOD1 and kinase RIP2 interact with bacterial peptidoglycan on early endosomes to promote autophagy and inflammatory signaling. Cell Host Microbe. (2014) 15:623–35. doi: 10.1016/j.chom.2014.04.001, PMID: 24746552

[38] Kaparakis M Turnbull L Carneiro L Firth S Coleman HA Parkington HC . Bacterial membrane vesicles deliver peptidoglycan to NOD1 in epithelial cells. Cell Microbiol. (2010) 12:372–85. doi: 10.1111/j.1462-5822.2009.01404.x, PMID: 19888989

[39] Kesty NC Mason KM Reedy M Miller SE Kuehn MJ . Enterotoxigenic Escherichia coli vesicles target toxin delivery into mammalian cells. EMBO J. (2004) 23:4538–49. doi: 10.1038/sj.emboj.7600471, PMID: 15549136 PMC533055

[40] Kadurugamuwa JL Beveridge TJ . Virulence factors are released from pseudomonas-aeruginosa in association with membrane-vesicles during normal growth and exposure to gentamicin - a novel mechanism of enzyme-secretion. J Bacteriol. (1995) 177:3998–4008. doi: 10.1128/jb.177.14.3998-4008.1995, PMID: 7608073 PMC177130

[41] Nice JB Collins SM Agro SMJ Sinani A Moros SD Pasch LM . Heterogeneity of size and toxin distribution in aggregatibacter actinomycetemcomitans outer membrane vesicles. Toxins. (2024) 16. doi: 10.3390/toxins16030138, PMID: 38535804 PMC10974469

[42] Hickey CA Kuhn KA Donermeyer DL Porter NT Jin C Cameron EA . Colitogenic Bacteroides thetaiotaomicron Antigens Access Host Immune Cells in a Sulfatase-Dependent Manner via Outer Membrane Vesicles. Cell Host Microbe. (2015) 17:672–80. doi: 10.1016/j.chom.2015.04.002, PMID: 25974305 PMC4432250

[43] Murase K Cytolysin A . (ClyA): A bacterial virulence factor with potential applications in nanopore technology, vaccine development, and tumor therapy. Toxins. (2022) 14:78. doi: 10.3390/toxins14020078, PMID: 35202106 PMC8880466

[44] Turnbull L Toyofuku M Hynen AL Kurosawa M Pessi G Petty NK . Explosive cell lysis as a mechanism for the biogenesis of bacterial membrane vesicles and biofilms. Nat Commun. (2016) 7:11220. doi: 10.1038/ncomms11220, PMID: 27075392 PMC4834629

[45] Bitto NJ Chapman R Pidot S Costin A Lo C Choi J . Bacterial membrane vesicles transport their DNA cargo into host cells. Sci Rep-Uk. (2017) 7:7072. doi: 10.1038/s41598-017-07288-4, PMID: 28765539 PMC5539193

[46] Dorward DW Garon CF Judd RC . Export and intercellular transfer of DNA via membrane blebs of neisseria-gonorrhoeae. J Bacteriol. (1989) 171:2499–505. doi: 10.1128/jb.171.5.2499-2505.1989, PMID: 2496108 PMC209926

[47] Johnston EL Zavan L Bitto NJ Petrovski S Hill AF Kaparakis-Liaskos M . Planktonic and biofilm-derived pseudomonas aeruginosa outer membrane vesicles facilitate horizontal gene transfer of plasmid DNA. Microbiol Spectr. (2023) 11:e0517922. doi: 10.1128/spectrum.05179-22, PMID: 36946779 PMC10100964

[48] Koeppen K Hampton TH Jarek M Scharfe M Gerber SA Mielcarz DW . A Novel Mechanism of Host-Pathogen Interaction through sRNA in Bacterial Outer Membrane Vesicles. PloS Pathog. (2016) 12:e1005672. doi: 10.1371/journal.ppat.1005672, PMID: 27295279 PMC4905634

[49] Ghosal A Upadhyaya BB Fritz JV Heintz-Buschart A Desai MS Yusuf D . The extracellular RNA complement of Escherichia coli. Microbiologyopen. (2015) 4:252–66. doi: 10.1002/mbo3.235, PMID: 25611733 PMC4398507

[50] Liu D Liu S Liu J Miao L Zhang S Pan Y . sRNA23392 packaged by porphyromonas gingivalis outer membrane vesicles promotes oral squamous cell carcinomas migration and invasion by targeting desmocollin-2. Mol Oral Microbiol. (2021) 36:182–91. doi: 10.1111/omi.12334, PMID: 33764008

[51] Guerrero-Mandujano A Hernández-Cortez C Ibarra JA Castro-Escarpulli G . The outer membrane vesicles: Secretion system type zero. Traffic. (2017) 18:425–32. doi: 10.1111/tra.12488, PMID: 28421662

[52] Mozaheb N Mingeot-Leclercq MP . Membrane vesicle production as a bacterial defense against stress. Front Microbiol. (2020) 11. doi: 10.3389/fmicb.2020.600221, PMID: 33362747 PMC7755613

[53] Huang WL Meng LX Chen Y Dong ZQ Peng Q . Bacterial outer membrane vesicles as potential biological nanomaterials for antibacterial therapy. Acta Biomater. (2022) 140:102–15. doi: 10.1016/j.actbio.2021.12.005, PMID: 34896632

[54] Lynch JB Schwartzman JA Bennett BD McAnulty SJ Knop M Nyholm SV . Ambient pH alters the protein content of outer membrane vesicles, driving host development in a beneficial symbiosis. J Bacteriol. (2019) 201:e00319–19. doi: 10.1128/JB.00319-19, PMID: 31331976 PMC6755730

[55] Baumgarten T Sperling S Seifert a Bergen Mv Steiniger F Wick LY . Membrane vesicle formation as a multiple-stress response mechanism enhances Pseudomonas putida DOT-T1E cell surface hydrophobicity and biofilm formation. Appl Environ Microb. (2012) 78:6217–24. doi: 10.1128/AEM.01525-12, PMID: 22752175 PMC3416621

[56] Manning AJ Kuehn MJ . Contribution of bacterial outer membrane vesicles to innate bacterial defense. BMC Microbiol. (2011) 11:258. doi: 10.1186/1471-2180-11-258, PMID: 22133164 PMC3248377

[57] Kulkarni HM Swamy CVB Jagannadham VM . Molecular characterization and functional analysis of outer membrane vesicles from the antarctic bacterium Pseudomonas syringae suggest a possible response to environmental conditions. J Proteome Res. (2014) 13:1345–58. doi: 10.1021/pr4009223, PMID: 24437924

[58] González LJ Bahr G Nakashige TG Nolan EM Bonomo RA Vila AJ . Membrane anchoring stabilizes and favors secretion of New Delhi metallo-β-lactamase. Nat Chem Biol. (2016) 12:516–+. doi: 10.1038/Nchembio.2083, PMID: 27182662 PMC4912412

[59] Marchant P Vivanco E Silva A Nevermann J Fuentes I Barrera B . β-lactam-induced OMV release promotes polymyxin tolerance in Salmonella enterica sv. Typhi. Front Microbiol. (2024) 15. doi: 10.3389/fmicb.2024.1389663, PMID: 38591031 PMC10999688

[60] Mashburn LM Whiteley M . Membrane vesicles traffic signals and facilitate group activities in a prokaryote. Nature. (2005) 437:422–5. doi: 10.1038/nature03925, PMID: 16163359

[61] Toyofuku M Morinaga K Hashimoto Y Uhl J Shimamura H Inaba H . Membrane vesicle-mediated bacterial communication. Isme J. (2017) 11:1504–9. doi: 10.1038/ismej.2017.13, PMID: 28282039 PMC5437348

[62] Brameyer S Plener L Müller A Klingl A Wanner G Jung K . Outer membrane vesicles facilitate trafficking of the hydrophobic signaling molecule CAI-1 between vibrio harveyi cells. J Bacteriol. (2018) 200:e00740–17. doi: 10.1128/JB.00740-17, PMID: 29555694 PMC6040191

[63] Li ZS Clarke AJ Beveridge TJ . A major autolysin of Pseudomonas aeruginosa: subcellular distribution, potential role in cell growth and division and secretion in surface membrane vesicles. J Bacteriol. (1996) 178:2479–88. doi: 10.1128/jb.178.9.2479-2488.1996, PMID: 8626312 PMC177969

[64] Evans AGL Davey HM Cookson A Currinn H Cooke-Fox G Stanczyk PJ . Predatory activity of Myxococcus xanthus outer-membrane vesicles and properties of their hydrolase cargo. Microbiology. (2012) 158:2742–52. doi: 10.1099/mic.0.060343-0, PMID: 22977088

[65] Clarke AJ . The “hole” story of predatory outer-membrane vesicles. Can J Microbiol. (2018) 64:589–99. doi: 10.1139/cjm-2017-0466, PMID: 30169125

[66] Fulsundar S Harms K Flaten GE Johnsen PJ Chopade BA Nielsen KM . Gene transfer potential of outer membrane vesicles of Acinetobacter baylyi and effects of stress on vesiculation. Appl Environ Microb. (2014) 80:3469–83. doi: 10.1128/AEM.04248-13, PMID: 24657872 PMC4018862

[67] Zingl FG Thapa HB Scharf M Kohl P Müller AM Schild S . Outer Membrane Vesicles of Vibrio cholerae Protect and Deliver Active Cholera Toxin to Host Cells via Porin-Dependent Uptake. Mbio. (2021) 12:e00534–21. doi: 10.1128/mBio.00534-21, PMID: 34076466 PMC8262896

[68] Choi JW Kim SC Hong SH Lee HJ . Secretable small RNAs via outer membrane vesicles in periodontal pathogens. J Dent Res. (2017) 96:458–66. doi: 10.1177/0022034516685071, PMID: 28068479

[69] Jang SC Kim SR Yoon YJ Park K-S Kim JH Lee J . *In vivo* kinetic biodistribution of nano-sized outer membrane vesicles derived from bacteria. Small. (2015) 11:456–61. doi: 10.1002/smll.201401803, PMID: 25196673

[70] Han E-C Choi S-Y Lee Y Park J-W Hong S-H Lee H-J . Extracellular RNAs in periodontopathogenic outer membrane vesicles promote TNF-α production in human macrophages and cross the blood-brain barrier in mice. FASEB J. (2019) 33:13412–22. doi: 10.1096/fj.201901575R, PMID: 31545910 PMC6894046

[71] O’Donoghue EJ Krachler AM . Mechanisms of outer membrane vesicle entry into host cells. Cell Microbiol. (2016) 18:1508–17. doi: 10.1111/cmi.12655, PMID: 27529760 PMC5091637

[72] O’Donoghue EJ Sirisaengtaksin N Browning DF Bielska E Hadis M Fernandez-Trillo F . Lipopolysaccharide structure impacts the entry kinetics of bacterial outer membrane vesicles into host cells. PloS Pathogen. (2017) 13:e1006760. doi: 10.1371/journal.ppat.1006760, PMID: 29186191 PMC5724897

[73] Elmi A Watson E Sandu P Gundogdu O Mills DC Inglis NF . Campylobacter jejuni outer membrane vesicles play an important role in bacterial interactions with human intestinal epithelial cells. Infect Immun. (2012) 80:4089–98. doi: 10.1128/IAI.00161-12, PMID: 22966047 PMC3497446

[74] Bachmann MF Jennings GT . Vaccine delivery: a matter of size, geometry, kinetics and molecular patterns. Nat Rev Immunol. (2010) 10:787–96. doi: 10.1038/nri2868, PMID: 20948547

[75] Liu Q Liu Q Yi J Liang K Liu T Roland KL . Outer membrane vesicles derived from Salmonella Typhimurium mutants with truncated LPS induce cross-protective immune responses against infection of Salmonella enterica serovars in the mouse model. Int J Med Microbiol. (2016) 306:697–706. doi: 10.1016/j.ijmm.2016.08.004, PMID: 27578609 PMC5206754

[76] Kim OY Park HT Dinh NTH Choi SJ Lee J Kim JH . Bacterial outer membrane vesicles suppress tumor by interferon-γ- mediated antitumor response. Nat Commun. (2017) 8:626. doi: 10.1038/s41467-017-00729-8, PMID: 28931823 PMC5606984

[77] Zhuang Q Xu J Deng D Chao T Li J Zhang R . Bacteria-derived membrane vesicles to advance targeted photothermal tumor ablation. Biomaterials. (2021) 268:120550. doi: 10.1016/j.biomaterials.2020.120550, PMID: 33278684

[78] Martins RM Maia MLS Camacho LAB Noronha TG von Doellinger VR Santos AP . Phase II/III randomized immunogenicity and safety study of a Brazilian meningococcal serogroup B vaccine in children. Vaccine. (2025) 61:127363. doi: 10.1016/j.vaccine.2025.127363, PMID: 40494223

[79] Marsay L Dold C Green CA Rollier CS Norheim G Sadarangani M . A novel meningococcal outer membrane vesicle vaccine with constitutive expression of FetA: A phase I clinical trial. J Infect. (2015) 71:326–37. doi: 10.1016/j.jinf.2015.05.006, PMID: 25982025 PMC4535279

[80] Katial RK Brandt BL Moran EE Marks S Agnello V Zollinger WD . Immunogenicity and safety testing of a group B intranasal meningococcal native outer membrane vesicle vaccine. Infect Immun. (2002) 70:702–7. doi: 10.1128/IAI.70.2.702-707.2002, PMID: 11796602 PMC127675

[81] Drabick JJ Brandt BL Moran EE Saunders NB Shoemaker DR Zollinger WD . Safety and immunogenicity testing of an intranasal group B meningococcal native outer membrane vesicle vaccine in healthy volunteers. Vaccine. (1999) 18:160–72. doi: 10.1016/S0264-410X(99)00216-9, PMID: 10501246

[82] Keiser PB Gibbs BT Coster TS Moran EE Stoddard MB Labrie JE 3rd . A phase 1 study of a group B meningococcal native outer membrane vesicle vaccine made from a strain with deleted lpxL2 and synX and stable expression of opcA. Vaccine. (2010) 28:6970–6. doi: 10.1016/j.vaccine.2010.08.048, PMID: 20732470

[83] Abara WE Bernstein KT Lewis FMT Schillinger JA Feemster K Pathela P . Effectiveness of a serogroup B outer membrane vesicle meningococcal vaccine against gonorrhoea: a retrospective observational study. Lancet Infect Dis. (2022) 22:1021–9. doi: 10.1016/S1473-3099(21)00812-4, PMID: 35427490 PMC10227473

[84] Johnson B . GSK’s gonorrhea vaccine receives fast-track designation to expedite clinical trials. Nat Med. (2023) 29:2146–7. doi: 10.1038/d41591-023-00069-9, PMID: 37550402

[85] Hu K Palmieri E Samnuan K Ricchetti B Oldrini D McKay PF . Generalized Modules for Membrane Antigens (GMMA), an outer membrane vesicle-based vaccine platform, for efficient viral antigen delivery. J Extracell Vesicles. (2022) 11:e12247. doi: 10.1002/jev2.12247, PMID: 36377074 PMC9663859

[86] Launay O Ndiaye AGW Conti V Loulergue P Sciré AS Landre AM . Booster vaccination with GVGH shigella sonnei 1790GAHB GMMA vaccine compared to single vaccination in unvaccinated healthy European adults: results from a phase 1 clinical trial. Front Immunol. (2019) 10:335. doi: 10.3389/fimmu.2019.00335, PMID: 30906291 PMC6418009

[87] Obiero CW Ndiaye AGW Sciré AS Kaunyangi BM Marchetti E Gone AM . A Phase 2a Randomized Study to Evaluate the Safety and Immunogenicity of the 1790GAHB Generalized Modules for Membrane Antigen Vaccine against Shigella sonnei Administered Intramuscularly to Adults from a Shigellosis-Endemic Country. Front Immunol. (2017) 8:1884. doi: 10.3389/fimmu.2017.01884, PMID: 29375556 PMC5763125

[88] Steimle A Autenrieth IB Frick JS . Structure and function: Lipid A modifications in commensals and pathogens. Int J Med Microbiol. (2016) 306:290–301. doi: 10.1016/j.ijmm.2016.03.001, PMID: 27009633

[89] Needham BD Trent MS . Fortifying the barrier: the impact of lipid A remodelling on bacterial pathogenesis. Nat Rev Microbiol. (2013) 11:467–81. doi: 10.1038/nrmicro3047, PMID: 23748343 PMC6913092

[90] Dovala D Rath CM Hu Q Sawyer WS Shia S Elling RA . Structure-guided enzymology of the lipid A acyltransferase LpxM reveals a dual activity mechanism. P Natl Acad Sci USA. (2016) 113:E6064–E71. doi: 10.1073/pnas.1610746113, PMID: 27681620 PMC5068295

[91] Ranallo RT Kaminski RW George T Kordis AA Chen Q Szabo K . Virulence, Inflammatory Potential, and Adaptive Immunity Induced by Shigella flexneri msbB Mutants. Infect Immun. (2010) 78:400–12. doi: 10.1128/IAI.00533-09, PMID: 19884336 PMC2798193

[92] Simpson BW Trent MS . Pushing the envelope: LPS modifications and their consequences. Nat Rev Microbiol. (2019) 17:403–16. doi: 10.1038/s41579-019-0201-x, PMID: 31142822 PMC6913091

[93] Wang X McGrath SC Cotter RJ Raetz CRH . Expression cloning and periplasmic orientation of the Francisella novicida lipid A 4’-phosphatase LpxF. J Biol Chem. (2006) 14:9321–30. doi: 10.1074/jbc.M600435200, PMID: 16467300 PMC2758525

[94] Wang X Karbarz MJ McGrath SC Cotter RJ Raetz CRH . MsbA transporter-dependent lipid A 1-dephosphorylation on the periplasmic surface of the inner membrane: topography of Francisella novicida LpxE expressed in Escherichia coli. J Biol Chem. (2004) 279:49470–8. doi: 10.1074/jbc.M409078200, PMID: 15339914 PMC2552400

[95] Bishop RE Gibbons HS Guina T Trent M Miller SI Raetz CR . Transfer of palmitate from phospholipids to lipid A in outer membranes of Gram-negative bacteria. EMBO J. (2000) 19:5071–80. doi: 10.1093/emboj/19.19.5071, PMID: 11013210 PMC302101

[96] Trent MS Pabich W Raetz CRH Miller SI . PhoP/PhoQ-induced lipase (PagL) that catalyzes 3-O-deacylation of lipid A precursors in membranes of Salmonella typhimurium. J Biol Chem. (2001) 276:9083–92. doi: 10.1074/jbc.M010730200, PMID: 11108722

[97] Gerritzen MJH Martens DE Wijffels RH Pol L Stork M . Bioengineering bacterial outer membrane vesicles as vaccine platform. Biotechnol Advances. (2017) 35:565–74. doi: 10.1016/j.biotechadv.2017.05.003, PMID: 28522212

[98] Gao W Fang RH Thamphiwatana S Luk BT Li J Angsantikul P . Modulating antibacterial immunity via bacterial membrane-coated nanoparticles. Nano Lett. (2015) 15:1403–9. doi: 10.1021/nl504798g, PMID: 25615236 PMC4399974

[99] Qin JQ Yang T Li JY Zhan GT Li X Wei ZH . Bacterial outer membrane vesicle-templated biomimetic nanoparticles for synergistic photothermo-immunotherapy. Nano Today. (2022) 46. doi: 10.1016/j.nantod.2022.101591

[100] Cai D Li Z Gao W Liu J Qi X Jin J . Proinflammatory macrophage-targeted nanoparticles rejuvenate aged macrophages and their phagocytic capacity. ACS Nano. (2025) 19:40002–40022. doi: 10.1021/acsnano.5c14201, PMID: 41220235

[101] Çelik PA Erdogan-Gover K Barut D Enuh BM Amasya G Sengel-Türk CT . Bacterial membrane vesicles as smart drug delivery and carrier systems: A new nanosystems tool for current anticancer and antimicrobial therapy. Pharmaceutics. (2023) 15:1052. doi: 10.3390/pharmaceutics15041052, PMID: 37111538 PMC10142793

[102] Alves NJ Turner KB Daniele MA Oh E Medintz IL Walper SA . Bacterial nanobioreactors–directing enzyme packaging into bacterial outer membrane vesicles. ACS Appl Mater Inter. (2015) 7:24963–72. doi: 10.1021/acsami.5b08811, PMID: 26479678

[103] Sepahdar Z Miroliaei M Bouzari S Khalaj V Salimi M . Surface engineering of escherichia coli–derived OMVs as promising nano-carriers to target EGFR-overexpressing breast cancer cells. Front Pharmacol. (2021) 12. doi: 10.3389/fphar.2021.719289, PMID: 34867325 PMC8638777

[104] Li Y Ma X Yue Y Zhang K Cheng K Feng Q . Rapid surface display of mRNA antigens by bacteria-derived outer membrane vesicles for a personalized tumor vaccine. Adv Mater. (2022) 34:e2109984. doi: 10.1002/adma.202109984, PMID: 35315546

[105] Wang H Zhan H Pan B Zeng L Chen Z Liu S . Engineering CRISPR system-based bacterial outer membrane vesicle potentiates T cell immunity for enhanced cancer immunotherapy. Adv Mater. (2025) 37:e2501565. doi: 10.1002/adma.202501565, PMID: 40495695 PMC12506603

[106] Levy M Kolodziejczyk AA Thaiss CA Elinav E . Dysbiosis and the immune system. Nat Rev Immunol. (2017) 17:219–32. doi: 10.1038/nri.2017.7, PMID: 28260787

[107] Bouffartigues E Gicquel G Bazire A Bains M Maillot O Vieillard J . Transcription of the gene of is dependent mainly on the SigX sigma factor and is sucrose induced. J Bacteriol. (2012) 194:4301–11. doi: 10.1128/JB.00509-12, PMID: 22685281 PMC3416264

[108] Cassin EK Tseng BS . Pushing beyond the envelope: the potential roles of OprF in biofilm formation and pathogenicity. J Bacteriol. (2019) 201:e00050–19. doi: 10.1128/JB.00050-19, PMID: 31010902 PMC6707909

[109] Wessel AK Liew J Kwon T Marcotte EM Whiteley M . Role of peptidoglycan-associated outer membrane proteins in vesicle formation. J Bacteriol. (2013) 195:213–9. doi: 10.1128/JB.01253-12, PMID: 23123904 PMC3553829

[110] Song T Mika F Lindmark B Liu Z Schild S Bishop A . A new Vibrio cholerae sRNA modulates colonization and affects release of outer membrane vesicles. Mol Microbiol. (2008) 70:100–11. doi: 10.1111/j.1365-2958.2008.06392.x, PMID: 18681937 PMC2628432

[111] Kulp A Kuehn MJ . Biological functions and biogenesis of secreted bacterial outer membrane vesicles. Annu Rev Microbiol. (2010) 64:163–84. doi: 10.1146/annurev.micro.091208.073413, PMID: 20825345 PMC3525469

[112] McBroom AJ Johnson AP Vemulapalli S Kuehn MJ . Outer membrane vesicle production by is independent of membrane instability. J Bacteriol. (2006) 188:5385–92. doi: 10.1128/JB.00498-06, PMID: 16855227 PMC1540050

[113] Freire P Vieira HLA Furtado AR Pedro M Arraiano CM . Effect of the morphogene on the permeability of the outer membrane. FEMS Microbiol Lett. (2006) 260:106–11. doi: 10.1111/j.1574-6968.2006.00307.x, PMID: 16790025

[114] Schwechheimer C Kuehn MJ . Synthetic effect between envelope stress and lack of outer membrane vesicle production in. J Bacteriol. (2013) 195:4161–73. doi: 10.1128/JB.02192-12, PMID: 23852867 PMC3754735

[115] Bardwell JC Lee JO Jander G Martin N Belin D Beckwith J . A pathway for disulfide bond formation *in vivo*. Proc Natl Acad Sci U.S.A. (1993) 90:1038–42. doi: 10.1073/pnas.90.3.1038, PMID: 8430071 PMC45806

[116] Bernadac A Gavioli M Lazzaroni J-C Raina S Lloubès R . Escherichia coli tol-pal Mutants Form Outer Membrane Vesicles. J Bacteriol. (1998) 180:4872–8. doi: 10.1128/JB.180.18.4872-4878.1998, PMID: 9733690 PMC107512

[117] Turner L Praszkier J Hutton ML Steer D Ramm G Kaparakis-Liaskos M . Increased outer membrane vesicle formation in a mutant. Helicobacter. (2015) 20:269–83. doi: 10.1111/hel.12196, PMID: 25669590

[118] Li Q Li Z Fei X Tian Y Zhou G Hu Y . The role of TolA, TolB, and TolR in cell morphology, OMVs production, and virulence of Choleraesuis. Amb Express. (2022) 12:5. doi: 10.1186/s13568-022-01347-4, PMID: 35075554 PMC8787014

[119] Cascales E Bernadac A Gavioli M Lazzaroni J-C Lloubes R . Pal lipoprotein of Escherichia coli plays a major role in outer membrane integrity. J Bacteriol. (2002) 184:754–9. doi: 10.1128/JB.184.3.754-759.2002, PMID: 11790745 PMC139529

[120] Rolhion N Barnich N Claret L Darfeuille-Michaud A . Strong decrease in invasive ability and outer membrane vesicle release in Crohn’s disease-associated adherent-invasive Escherichia coli strain LF82 with the yfgL gene deleted. J Bacteriol. (2005) 187:2286–96. doi: 10.1128/JB.187.7.2286-2296.2005, PMID: 15774871 PMC1065249

[121] Ojima Y Sawabe T Konami K Azuma M . Construction of hypervesiculation Escherichia coli strains and application for secretory protein production. Biotechnol Bioeng. (2020) 117:701–9. doi: 10.1002/bit.27239, PMID: 31788781

[122] Kim DY . Two stress sensor proteins for the expression of sigmaE regulon: DegS and RseB. J Microbiol. (2015) 53:306–10. doi: 10.1007/s12275-015-5112-6, PMID: 25935301

[123] Marinacci B Krzyzek P Pellegrini B Turacchio G Grande R . Latest update on outer membrane vesicles and their role in horizontal gene transfer: A mini-review. Membranes-Basel. (2023) 13:860. doi: 10.3390/membranes13110860, PMID: 37999346 PMC10673008

[124] Murase K Martin P Porcheron G Houle S Helloin E Pénary M . HlyF produced by extraintestinal pathogenic is a virulence factor that regulates outer membrane vesicle biogenesis. J Infect Dis. (2016) 213:856–65. doi: 10.1093/infdis/jiv506, PMID: 26494774

[125] Goman A Ize B Jeannot K Pin C Payros D Goursat C . Uncovering a new family of conserved virulence factors that promote the production of host-damaging outer membrane vesicles in gram-negative bacteria. J Extracell Vesicles. (2025) 14:e270032. doi: 10.1002/jev2.70032, PMID: 39840902 PMC11752146

[126] Kitagawa R Takaya A Ohya M Mizunoe Y Takade A Yoshida S-i . Biogenesis of serovar typhimurium membrane vesicles provoked by induction of pagC. J Bacteriol. (2010) 192:5645–56. doi: 10.1128/JB.00590-10, PMID: 20802043 PMC2953678

[127] Arnold T Poynor M Nussberger S Lupas AN Linke D . Gene duplication of the eight-stranded β-barrel OmpX produces a functional pore:: A scenario for the evolution of transmembrane β-barrels. J Mol Biol. (2007) 366:1174–84. doi: 10.1016/j.jmb.2006.12.029, PMID: 17217961

[128] Schwechheimer C Kuehn MJ . Synthetic effect between envelope stress and lack of outer membrane vesicle production in Escherichia coli. J Bacteriol. (2013) 195:4161–73. doi: 10.1128/JB.02192-12, PMID: 23852867 PMC3754735

[129] Choi H-I Kim M Jeon J Han JK Kim K-S . Overexpression of MicA induces production of OmpC-enriched outer membrane vesicles that protect against challenge. Biochem Bioph Res Co. (2017) 490:991–6. doi: 10.1016/j.bbrc.2017.06.152, PMID: 28666873

[130] Schwechheimer C Rodriguez DL Kuehn MJ . NlpI-mediated modulation of outer membrane vesicle production through peptidoglycan dynamics in Escherichia coli. Microbiologyopen. (2015) 4:375–89. doi: 10.1002/mbo3.244, PMID: 25755088 PMC4475382

[131] Chen DJ Osterrieder N Metzger SM Buckles E Doody AM DeLisa MP . Delivery of foreign antigens by engineered outer membrane vesicle vaccines. Proc Natl Acad Sci U S A. (2010) 107:3099–104. doi: 10.1073/pnas.0805532107, PMID: 20133740 PMC2840271

[132] Nie D Hu Y Chen Z Li MK Hou Z Luo XX . Outer membrane protein A (OmpA) as a potential therapeutic target for Acinetobacter baumannii infection. J BioMed Sci. (2020) 27:26. doi: 10.1186/s12929-020-0617-7, PMID: 31954394 PMC6969976

[133] Jin ML Huo D Sun JJ Hu JC Liu SZ Zhan MS . Enhancing immune responses of ESC-based TAA cancer vaccines with a novel OMV delivery system. J Nanobiotechnol. (2024) 22:15. doi: 10.1186/s12951-023-02273-8, PMID: 38166929 PMC10763241

[134] Gallus S Peschke T Paulsen M Burgahn T Niemeyer CM Rabe KS . Surface display of complex enzymes by SpyCatcher-SpyTag interaction. Chembiochem. (2020) 21:2126–31. doi: 10.1002/cbic.202000102, PMID: 32182402 PMC7497234

[135] Laotee S Arunmanee W . Genetically surface-modified Escherichia coli outer membrane vesicles targeting MUC1 antigen in cancer cells. Biotechnol Rep. (2024) 3:e00854. doi: 10.1016/j.btre.2024.e00854, PMID: 39290790 PMC11406022

[136] Jong WSP Soprova Z Punder Kd Hagen-Jongman C Wagner S Wickström D . A structurally informed autotransporter platform for efficient heterologous protein secretion and display. Microb Cell Factories. (2012) 11:85. doi: 10.1186/1475-2859-11-85, PMID: 22709508 PMC3521207

[137] Daleke-Schermerhorn MH Felix T Soprova Z Hagen-Jongman CMT Vikström D Majlessi L . Decoration of outer membrane vesicles with multiple antigens by using an autotransporter approach. Appl Environ Microb. (2014) 80:5854–65. doi: 10.1128/AEM.01941-14, PMID: 25038093 PMC4178611

[138] Kuipers K SchermerhornJong MHD Jong WSP Hagen-Jongman C Opzeeland Fv Simonetti E . Salmonella outer membrane vesicles displaying high densities of pneumococcal antigen at the surface offer protection against colonization. Vaccine. (2015) 33:2022–9. doi: 10.1016/j.vaccine.2015.03.010, PMID: 25776921

[139] Ninyio N Schmitt K Sergon G Nilsson C Andersson S Scherbak N . Stable expression of HIV-1 MPER extended epitope on the surface of the recombinant probiotic bacteria Nissle 1917 using CRISPR/Cas9. Microb Cell Factories. (2024) 23:39. doi: 10.1186/s12934-023-02290-0, PMID: 38311724 PMC10840157

[140] Han MJ . Novel bacterial surface display system based on the protein MipA. J Microbiol Biotechnol. (2020) 30:1097–103. doi: 10.4014/jmb.2001.01053, PMID: 32325544 PMC9728377

[141] Kothary MH Gopinath GR Gangiredla J Rallabhandi PV Harrison LM Yan QQ . Analysis and characterization of proteins associated with outer membrane vesicles secreted by cronobacter spp. Front Microbiol. (2017) 8:1664–302X. (Print). doi: 10.3389/fmicb.2017.00134, PMID: 28232819 PMC5299011

[142] Ureta AR Endres RG Wingreen NS Silhavy TJ . Kinetic analysis of the assembly of the outer membrane protein LamB in mutants each lacking a secretion or targeting factor in a different cellular compartment. J Bacteriol. (2007) 189:446–54. doi: 10.1128/JB.01103-06, PMID: 17071751 PMC1797403

[143] Georgiou G Stathopoulos C Daugherty PS Nayak AR Iverson BL III RC . Display of heterologous proteins on the surface of microorganisms: From the screening of combinatorial libraries to live recombinant vaccines. Nat Biotechnol. (1997) 15:29–34. doi: 10.1038/nbt0197-29, PMID: 9035102

[144] Kesty NC Kuehn MJ . Incorporation of heterologous outer membrane and periplasmic proteins into outer membrane vesicles. J Biol Chem. (2004) 279:2069–76. doi: 10.1074/jbc.M307628200, PMID: 14578354 PMC3525464

[145] Ojima Y Yamaguchi K Taya M . Quantitative evaluation of recombinant protein packaged into outer membrane vesicles of cells. Biotechnol Progr. (2018) 34:51–7. doi: 10.1002/btpr.2536, PMID: 28786214

[146] Taschner S Meinke A Gabain Av Boyd AP . Selection of peptide entry motifs by bacterial surface display. Biochem J. (2002) 367:393–402. doi: 10.1042/bj20020164, PMID: 12144529 PMC1222908

[147] Sauer DA-O Markel UA-O Schiffels JA-O Okuda JA-O Schwaneberg UA-OX . FhuA: from iron-transporting transmembrane protein to versatile scaffolds through protein engineering. Accounts Chem Res. (2023) 20:1433–44. doi: 10.1021/acs.accounts.3c00060, PMID: 37191525

[148] Garling A Goursat C Seguy C Martin P Goman A Nougayrède JP . Development of intimin-enriched outer membrane vesicles (OMVs) as a vaccine to control intestinal carriage of Enterohemorrhagic. Vaccine. (2025) 52:126899. doi: 10.1016/j.vaccine.2025.126899, PMID: 39985970

[149] Cho Y-H Kim S-J Kim J-Y Lee D-H Park K Park Y-C . Effect of PelB signal sequences on Pfe1 expression and ω-hydroxyundec-9-enoic acid biotransformation in recombinant Escherichia coli. Appl Microbiol Biotechnol. (2018) 102:7407–16. doi: 10.1007/s00253-018-9139-6, PMID: 29936545

[150] Hanumunthadu B Kanji N Owino N Ferreira Da Silva C Robinson H White R . Salmonella Vaccine Study in Oxford (SALVO) trial: protocol for an observer-participant blind randomised placebo-controlled trial of the iNTS-GMMA vaccine within a European cohort. BMJ Open. (2023) 13:e072938. doi: 10.1136/bmjopen-2023-072938, PMID: 37963701 PMC10649500

[151] Skidmore PD Canals R Ramasamy MN . The iNTS-GMMA vaccine: a promising step in non-typhoidal Salmonella vaccine development. Expert Rev Vaccines. (2023) 22:918–20. doi: 10.1080/14760584.2023.2270596, PMID: 37824701

